# Bacterial partition complexes segregate within the volume of the nucleoid

**DOI:** 10.1038/ncomms12107

**Published:** 2016-07-05

**Authors:** Antoine Le Gall, Diego I. Cattoni, Baptiste Guilhas, Céline Mathieu-Demazière, Laura Oudjedi, Jean-Bernard Fiche, Jérôme Rech, Sara Abrahamsson, Heath Murray, Jean-Yves Bouet, Marcelo Nollmann

**Affiliations:** 1 Centre de Biochimie Structurale, CNRS UMR5048, INSERM U1054, Université de Montpellier, 29 rue de Navacelles, 34090 Montpellier, France; 2 Laboratoire de Microbiologie et Génétique Moléculaires, Centre de Biologie Intégrative, Centre National de la Recherche Scientifique, Université de Toulouse, UPS, F-31000 Toulouse, France; 3 HHMI, Lulu and Anthony Wang Laboratory of Neural Circuits and Behavior, The Rockefeller University, New York, New York 10065, USA; 4 Centre for Bacterial Cell Biology, Institute for Cell and Molecular Biosciences, Newcastle University, Newcastle Upon Tyne NE2 4AX, UK

## Abstract

Precise and rapid DNA segregation is required for proper inheritance of genetic material. In most bacteria and archaea, this process is assured by a broadly conserved mitotic-like apparatus in which a NTPase (ParA) displaces the partition complex. Competing observations and models imply starkly different 3D localization patterns of the components of the partition machinery during segregation. Here we use super-resolution microscopies to localize in 3D each component of the segregation apparatus with respect to the bacterial chromosome. We show that Par proteins locate within the nucleoid volume and reveal that proper volumetric localization and segregation of partition complexes requires ATPase and DNA-binding activities of ParA. Finally, we find that the localization patterns of the different components of the partition system highly correlate with dense chromosomal regions. We propose a new mechanism in which the nucleoid provides a scaffold to guide the proper segregation of partition complexes.

## Abstract (web summary)

In most bacteria and archaea, a broadly conserved mitotic-like apparatus assures the inheritance of duplicated genetic material before cell division. Here, the authors use super-resolution microscopies to dissect the activities required for proper DNA segregation through the nucleoid interior.

## Graphical abstract

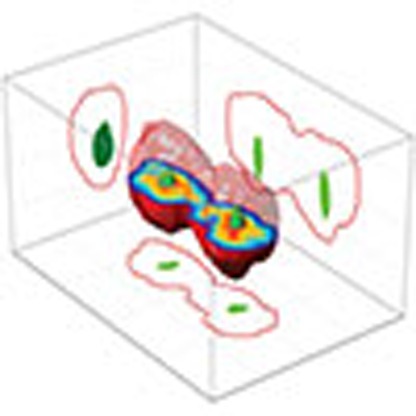

The preservation of all living forms relies on the faithful segregation of their duplicated genetic material upon cell division. To assure the relocalization of replicated DNA, prokaryotic organisms have evolved dedicated apparatuses, namely partition systems (Par), that are classified according to the nature of the ATPase providing the energy to displace DNA^1^,^2^. In particular, Type I Par systems are the most prevalent in low copy-number plasmids and the only type known to be present on bacterial chromosomes^3^. It contains three essential elements: a repeat of centromere-like DNA sequences (*parS*), a DNA-binding protein (ParB) and a deviant Walker A-type ATPase (ParA)^2^,^4^. ParB specifically recognizes *parS* and, upon binding, forms the partition complex (ParB*S*)^5^,^6^,^7^,^8^ that will be displaced by the action of ParA. The partition systems of plasmids F and P1 in *Escherichia coli*, and of the chromosome of *Bacillus subtilis*, are among the best characterized^9^.

Different models have been proposed to account for the assembly of partition complexes and for the role of ParA in their segregation. At physiological levels, ParB binds over a large genomic region flanking *parS* (10–12 kb). This extended binding pattern has been interpreted as resulting from the one-dimensional spreading of ParB from *parS*^10^,^11^,^12^, and more recently because of the dynamic stochastic binding and unbinding of ParB around *parS*^13^. Partition complexes are initially positioned at midcell and segregate to their new subcellular locations after or concomitantly with replication^1^. Importantly, proper segregation of partition complexes requires the ParB-stimulated ATPase activity of ParA as well as ParA's ability to bind nonspecific DNA (ns-DNA). Altering these activities leads to different degrees of plasmid instability and chromosome segregation defects^14^,^15^,^16^,^17^,^18^.

Two families of models have been developed to explain the mechanism by which ParA segregates partition complexes. The first class of models (‘filament' models) proposes that dynamic ParA filaments pull partition complexes to their resting positions, either around or straight through the nucleoid (Fig. 1a, upper panel)^19^,^20^. The second class of models (‘non-filament' models) involves a diffusion-ratchet mechanism (‘brownian-ratchet' model) in which ParA dimers or short filaments push–pull partition complexes on the surface of the nucleoid (Fig. 1a, lower panel)^21^,^22^,^23^,^24^. A variation of this mechanism (‘DNA relay' model) was recently proposed in which chromosome elasticity also plays a role by partly powering movement of the partition complex^25^ (Fig. 1a, lower panel). All these models were largely based on biochemical assays (for example, polymerization, DNA binding and ATPase activity assays), two-dimensional (2D) imaging and *in vitro* microscopy assays monitoring the dynamics of ParB-bound plasmids on a 2D ParA-bound DNA carpet, and proposed that partition components segregate through the space between the nucleoid surface and the membrane. In brief, these various models proposed distinct localization patterns of Par components within or around the nucleoid. Importantly, these patterns constitute essential ingredients of the proposed mechanisms. Discerning between these models requires the knowledge, *in vivo*, of the precise three-dimensional (3D) localization of the machinery components, yet so far this has been limited by the optical resolution of conventional microscopy methods.

Here we combine whole-chromosome labelling strategies with multicolour 3D super-resolution technologies to investigate the segregation machineries of plasmid F and the *B. subtilis* chromosome. Our strategy allowed us to reveal that partition complexes as well as the ParA ATPase reside within the nucleoid interior. By functional studies, we determine that both Walker-type ATPase and dynamic DNA-binding activities of ParA are necessary for proper volumetric relocalization of partition complexes during segregation. Finally, we unveil that both partition complexes and ParA tend to colocalize with high-density chromosomal regions (HDRs), suggesting a new mechanism by which chromosomal DNA may play a role by locally enriching components of the segregation apparatus to promote proper segregation.

## Results

### Partition complexes reside inside the bacterial nucleoid

We first implemented an experimental strategy to directly detect the 3D localization of partition complexes within the volume of the nucleoid. To this end, we combined high-density whole-chromosome labelling with high-throughput, 3D structured illumination microscopy (3D-SIM)^26^, a super-resolution method that provides a twofold increase in both lateral and axial resolution as compared with diffraction-limited microscopies. DNA was either fluorescently labelled by the ns-DNA-binding protein histone-like U93 (HU) tagged with monomeric fluorescent protein mCherry (nucleoids in *E. coli* strains) or by the intercalating agent 4,6-diamidino-2-phenylindole (nucleoids in *B. subtilis* strains), while localization of partition complexes was detected by tagging ParB with a fluorescent tag. ParB from the F-plasmid (ParB_F_ or SopB) was tagged with monomeric Venus (mVenus) at the locus under the control of the endogenous autoregulated promoter, while *B. subtilis* ParB (ParB_Bsu_ or Spo0J) was tagged using monomeric green fluorescent protein (GFP) also at its natural locus (see Supplementary Methods).

**Figure 1:**
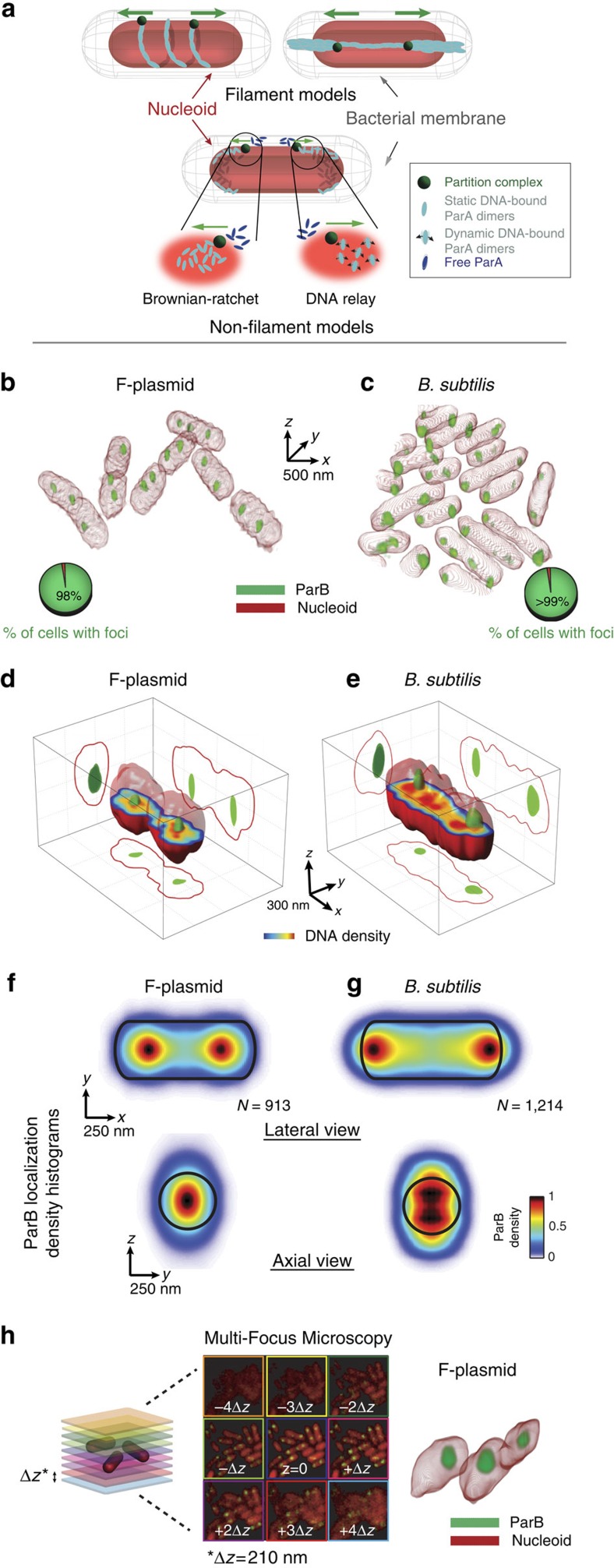
Partition complexes segregate through the interior of the bacterial nucleoid. (**a**) Schematic representation of filament (upper panel) and Brownian-ratchet (lower panel) models for active bacterial DNA segregation. In Brownian-ratchet models, partition complexes are segregated by a pure ParA diffusion-ratchet (lower panel left) or combined with the propulsion provided by the elastic properties of the chromosome (lower panel right). (**b**,**c**) Typical multicolour 3D-SIM images of (**a**) HU-mCherry-labelled nucleoids (red) and plasmidic ParB_F_-mVenus (green) in *E. coli* (DLT3053/pJYB234), or (**b**) 4,6-diamidino-2-phenylindole-stained nucleoids (red) and chromosomal ParB_Bsu_-GFP (green) in *B. subtilis* (HM671). (**d**,**e**) Characteristic 3D volume of single nucleoids (solid red) and ParB foci (green) with orthogonal 2D projections of nucleoid (red contour) and ParB (green spot) densities for (**d**) ParB_F_-mVenus and (**e**) ParB_Bsu_-GFP. (**f**,**g**) 2D density distributions of ParB intensity signal along the *xy* (lateral) and *yz* (axial) planes for (**f**) *E. coli* and (**g**) *B. subtilis* strains. Only nucleoids of equal length were used for calculation of ParB densities (see Methods for details). Nucleoid contours are represented with solid black lines with capsid shapes. Dimensions of nucleoid contours were measured as the full-width at half-maximum of the intensity signal along each nucleoid dimension. *N* depicts the number of nucleoids analysed. All panels of the figure represent one representative experiment of at least six biological replicates. (**h**) Instantaneous 3D MFM of ParB_F_ and nucleoids labelled with HU-mCherry. Left: MFM scheme representing the simultaneously acquired nine *z* planes for fast volumetric imaging. Middle: single processed image in which the nine simultaneously imaged *z* planes can be visualized at once after correction of chromatic aberrations (see Supplementary Fig. 4 and Supplementary Methods). Right: volumetric reconstruction of bacterial nucleoid (red) and ParB_F_ (green) from image in the left panel (only two nucleoids are shown for clarity).

We simultaneously imaged the bacterial chromosome and plasmidic/chromosomal ParB by 3D-SIM. *E. coli* nucleoids displayed a range of sizes between 800 and 4,000 nm in length and 530±50 nm in width (full-width at half-maximum (FWHM); Supplementary Fig. 1A,B). ParB foci were observed in a large proportion of cells (∼98%; Fig. 1b,c, and pie charts therein), consistent with the fluorescent tag not affecting plasmid stability^13^ or chromosome segregation^26^. The number of partition complexes per nucleoid (2.2±0.8, mean±s.d., Supplementary Fig. 1C) agreed well with the distributions observed using widefield microscopy or photo-activated localization microscopy (PALM)^13^. Partition complexes had a typical size of 140±35 nm (Supplementary Fig. 2), consistent with the sizes of partition complexes recently reported using PALM (∼150 nm)^13^.

Importantly, partition complexes were confined within the bacterial nucleoid for both plasmidic and chromosomal systems (Fig. 1b,c), consistent with preliminary observations from *B. subtilis* ParB^26^. Single projections of isolevel representations of 3D densities can often be misleading. Thus, we developed an algorithm to simultaneously visualize both colours (that is, nucleoid and ParB) in 3D as well as the three orthogonal 2D projections at the single-cell level. This visualization clearly reveals that ParB is localized within the bacterial nucleoid, and is particularly close to the centre of the nucleoid long axis (Fig. 1d,e, Supplementary Movies 1 and 2 and Supplementary Fig. 3). To determine whether this localization pattern was observed at the population level, we built localization density histograms of the positions of partition complexes within nucleoids from thousands of cells (see Methods for details). Figure 1f,g displays the projection of the cumulated localization of partition complexes for nucleoids of similar sizes (<1,500 nm in length) in 2D representations (*xy* and *yz* planes). Lateral histograms (*xy* plane) revealed that both plasmidic and chromosomal partition complexes are positioned along the longitudinal axis (Fig. 1f,g). ParB*S*_F_ partition complexes localized to the quarter nucleoid positions, while ParB*S*_Bsu_ complexes were positioned close to the nucleoid edges, as recently described^26^,^27^. Importantly, axial histograms (*yz* plane) revealed that ParB_F_ and ParB_Bsu_ complexes were also radially confined to the centre of the nucleoid axis (Fig. 1f,g, lower panels) but with a larger spread with respect to the nucleoid volume because of the lower axial resolution of 3D-SIM.

Acquisition of a single two-colour volume in 3D-SIM requires ∼20–50 s and involves complex image-reconstruction algorithms. Thus, we used a second advanced microscopy method to discard any potential image-blurring effects caused by the movement of the nucleoid and partition complexes during the acquisition time or any image-reconstruction artefact. Multifocus microscopy (MFM)^28^ allows for multicolour, near-instantaneous volumetric acquisitions (∼25 ms per channel) of biological specimens. Particularly, MFM permits the simultaneous acquisition of nine imaging planes and thus the volumetric acquisitions of entire nucleoids and partition complexes in a single snapshot without resorting to complex image-reconstruction algorithms (Fig. 1h, left and middle panels and Supplementary Fig. 4). Simultaneous MFM imaging of nucleoids and ParB_F_ resulted in equivalent results to those obtained by 3D-SIM (Fig. 1h, right panel).

Overall, these results demonstrate that the localization of partition complexes does not require interactions with the bacterial nucleoid surface. However, in that case, how can the ParA ATPase interact with ParB nucleoprotein complexes positioned inside the nucleoid?

### ParA displaces ParB within the bacterial nucleoid

**Figure 2:**
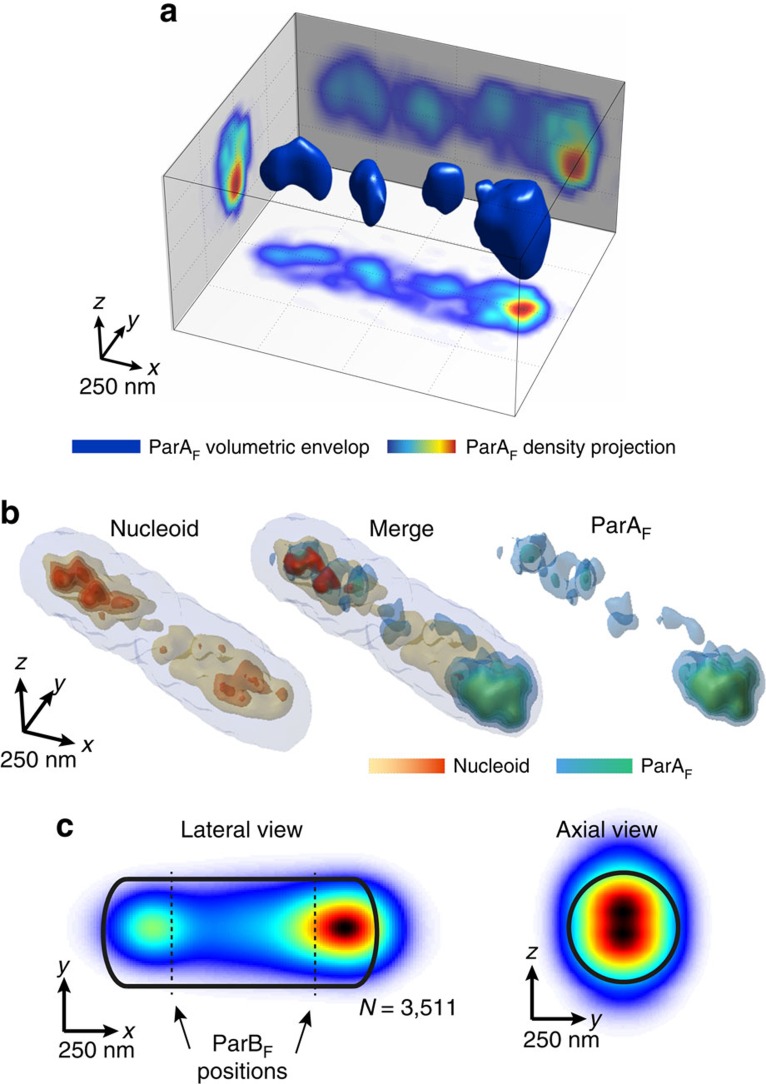
ParA is localized within the nucleoid volume. (**a**) 3D-SIM image from DLT3053/pJYB243 showing ParA_F_ (solid blue) with side projections of ParA_F_ densities (colour-coded scale for projections (a.u.) represents fluorescence intensity increasing from blue to red). (**b**) Typical two-colour, 3D-SIM images of a single nucleoid labelled with HU-mCherry and the ParA_F_-mVenus distribution at its interior. Left: nucleoid periphery is shown in light grey, while higher-density regions at the centre of the nucleoid are shown in orange-red. Right: ParA_F_ is represented in blue-green. Middle: overlay of the two colours showing the positioning of ParA_F_ relative to the nucleoid. (**c**) 2D distribution of ParA along the lateral (*xy*) and axial (*yz*) planes of the nucleoids. Nucleoid orientation is aligned from the brightest ParA_F_ patch. *N* depicts the number of cells analysed. All panels of the figure depict one representative experiment of at least five biological replicates.

Previous *in vitro* and 2D microscopy studies have led to a model in which ParA forms filaments (Fig. 1a)^19^,^20^,^29^. To test this model, we imaged ParA_F_-mVenus using 3D-SIM. We observed that ParA_F_ spreads into gradient-like, asymmetric structures with higher density of protein at one nucleoid pole (Fig. 2 and Supplementary Movie 3). The lateral and axial projections of the 3D volume of ParA_F_ revealed smaller and dimmer fluorescent patches distributed along the ParA_F_ density (Fig. 2a). To determine whether these asymmetric, discontinuous ParA structures distributed over the membrane/nucleoid surface or rather penetrated the nucleoid volume, we imaged ParA_F_-mVenus and nucleoid simultaneously by multicolour 3D-SIM (Fig. 2b and Supplementary Movie 4). Strikingly, ParA_F_ was distributed within the interior of the nucleoid extending asymmetrically along the long cell axis. 2D lateral histograms of ParA localization (*xy* plane) were constructed as previously described but aligning ParA images from different nucleoids to orient the highest density of ParA specifically on the right side of the histogram (Fig. 2c). The region with highest ParA density resided well within the nucleoid volume, and localized close to the quarter nucleoid positions displayed by ParB complexes (Fig. 2c, left panel, dotted lines). Interestingly, a second weaker ParA_F_ density maximum emerged near the opposite ParB_F_ quarter nucleoid position (Fig. 2c, left panel, compare with Fig. 1f). Axial histograms (*yz* plane) recapitulated well the volumetric distribution of ParA_F_. This distribution was radially confined to the longitudinal axis (as that of ParB_F_), but was more broadly distributed (Fig. 2c, right panel).

### ParA regulates the volumetric localization of ParBS

**Figure 3:**
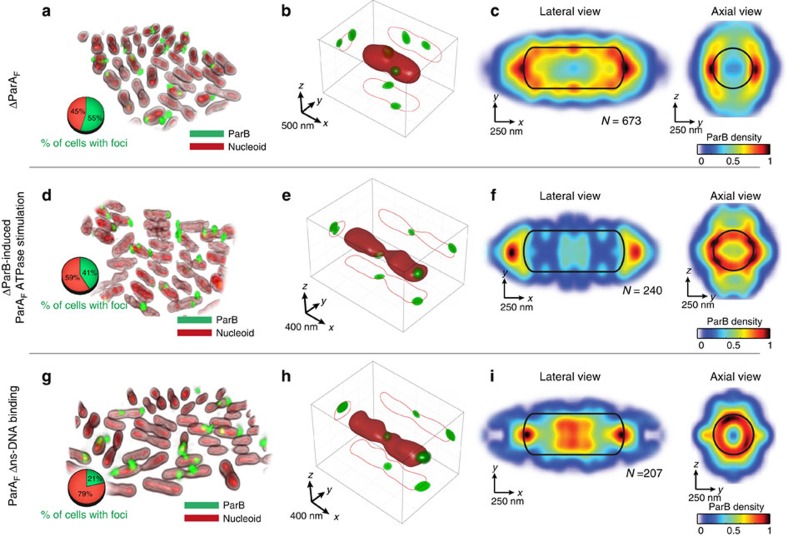
ParA_F_ determines the 3D localization of partition complexes within bacterial nucleoids. (**a**–**g**) Widefield deconvolution imaging of *E. coli* cells harbouring F-plasmid (**a**) lacking ParA_F_ (DLT3053/pJYB263; **d**) with ParA_F_^K120Q^ (DLT3053/pJYB277) and (**g**) with ParA_F_^K340A^, an allele with reduced ns-DNA-binding capacity (DLT3053/pJYB279). Representative fields of view of two-colour widefield deconvolution imaging of HU-mCherry-labelled nucleoids (red) and ParB_F_-mVenus (green) are shown for each of the mutants. Pie charts represent the percentage of nucleoids with (green) and without (red) ParB_F_ foci, reflecting the frequency of plasmid loss in these mutants. (**b**–**h**) Characteristic 3D volume of single nucleoids (solid red) and ParB_F_ foci (green) with orthogonal 2D projections of nucleoid (red contours) and ParB_F_ densities (green spots) for the three mutants shown in **a**–**g**. (**c**–**i**) 2D density distributions of ParB_F_ intensity signal along the *xy* (lateral) and *yz* (axial) planes for mutants displayed in **a**–**g**. Solid black lines represent nucleoid periphery. See legend of Fig. 1f,g and Methods for more details. Residual ParB_F_ density at midcell in **h**–**i** arises from ParB_F_ at the centre of nucleoids about to split (see examples in **e**,**f**). *N* represents number of cells. All panels of the figure depict one representative experiment of at least two biological replicates.

These results suggest that the nucleoid scaffold itself or the interaction between partition complexes and ParA could be responsible for the intranucleoid localization of the components of the segregation machinery. To functionally test these hypotheses, we evaluated the subnucleoid localization of partition complexes in absence of ParA_F_ and for specific ParA_F_ mutants known to affect DNA binding and ATP hydrolysis activities. First, *E. coli* cells carrying the ParB*S*_F_ system but not ParA_F_ were grown with antibiotics to maintain the F-plasmid. In absence of ParA_F_, partition complexes displayed a very dynamic behaviour that could not be captured using 3D-SIM. As the localization of partition complexes in wild-type cells is equivalent when imaged either by 3D-SIM or widefield deconvolution microscopy (Supplementary Fig. 5), we turned to the latter technique to facilitate the detection of partition complexes in mutant strains. About 55% of cells showed distinct bright ParB_F_ foci compared with >98% of cells carrying the wild-type ParAB*S* partition system (Figs 1b and 3a). This finding is expected from the plasmid loss rate observed without ParA_F_ (Supplementary Table 1). Volumetric views of single nucleoids reveal that most ParB_F_ foci were now located outside the nucleoid (Fig. 3b and Supplementary Movies 5, 6). These single-cell observations were recapitulated at the population level by calculating the lateral and axial histograms of ParB localization. ParB_F_ was preferentially located at the nucleoid periphery, while the nucleoid interior was mostly devoid of ParB density (Fig. 3c). These results indicate a key role of ParA_F_ in the localization of the partition complex within the nucleoid volume.

We next investigated how two key activities of ParA_F_—ATPase stimulation by ParB_F_ and ns-DNA binding—regulate the localization of partition complexes by employing ParA_F_ mutants. ParA_F_^K120Q^ retains wild-type basal ATPase hydrolysis rate; however, its ATPase activity is no longer stimulated by ParB_F_^30^. In contrast, ParA_F_^K340A^ retains its ability to interact with ParB_F_ but is partially impaired at ns-DNA-binding^14^,^18^. The percentage of cells displaying ParB_F_ foci was reduced from 98% (wild type) to 41% and 21% in the mutants (ParA_F_^K120Q^ and ParA_F_^K340A^, respectively, pie charts in Fig. 3d–g, and Supplementary Table 1).

Partition complexes localized outside the nucleoid volume for both ParA_F_ mutants (Fig. 3e–h and Supplementary Movies 7, 8, 9) and tended to localize to regions displaying less spatial confinement (that is, near cell poles and at the locations of future division septa), as previously shown for large protein complexes or plasmids without partitioning systems^31^,^32^. To validate these results at the population level, we calculated the mean localization of partition complexes within nucleoids (Fig. 3f–i). For ParA_F_^K120Q^, lateral (XY) ParB_F_ density histograms (Fig. 3f) displayed a similar pattern of localization than that of cells lacking ParA_F_ (Fig. 3c), with partition complexes clearly locating at the exterior of the nucleoid. ParA_F_^K340A^ also exhibited abnormal distributions of ParB_F_ but with a more heterogeneous distribution (Fig. 3i) likely because of residual interactions between ParA_F_^K340A^ and ParB_F_ as well as with ns-DNA. Interestingly, axial ParB_F_ density histograms (*yz*) for both mutants showed a disturbed radial profile in which ParB_F_ localized mostly to the edges of nucleoids (Fig. 3f–i, right panels). These results indicate that proper volumetric localization of partition complexes requires ns-DNA binding by ParA as well as the stimulation of ParA's ATPase activity by ParB.

### The partition machinery localizes to high-density regions

We recently described the existence of a new structural feature of the bacterial nucleoid of *B. subtilis*: HDRs^26^. HDRs represent regions of the nucleoid with denser DNA content and only resolvable by super-resolution microscopies. As both ParB and ParA distributed within the nucleoid volume, we evaluated whether HDRs played a role in their localization.

**Figure 4:**
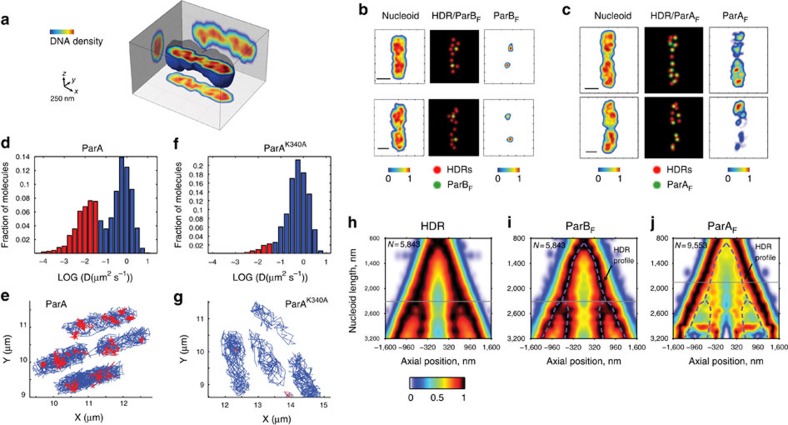
Partition complexes segregate through dense chromosomal DNA regions. (**a**) Typical 3D volume of chromosomal DNA stained with HU-mCherry. Nucleoid DNA density in the volumetric representation and side projections is colour-coded from blue to red. HDRs stretch from pole to pole and are joined by a semicontinuous filamentous density. (**b**) ParB_F_ localization correlates with positions occupied by HDRs. Left: maximum intensity projections of individual nucleoids labelled by HU-mCherry (DLT3053/pJYB234). Right: maximum intensity projections of the same nucleoids but for ParB_F_-mVenus. Middle: 2D projection of HDRs (red) and ParB_F_ foci (green) positions represented as 2D Gaussian distributions with sizes corresponding to the resolution of 3D-SIM for each emission channel. Note that this image represents a 2D projection, not a *z* section. HDRs colocalizing with ParB foci are shown in yellow. (**c**) ParA_F_ localization correlates with positions occupied by HDRs. Same representations as in **b** but for HDRs and ParA_F_ (DLT3053/pJYB243). A strong correlation between HDR and ParA is observed despite the nucleoid and ParAB dynamics between sequential acquisitions for each colour. (**d**–**f**) Distributions of the apparent diffusion coefficient, *D*, of single-molecule trajectories of wild-type ParA (DLT3284/pJYB286, *N*=63 cells) and ParAF^K340A^ (DLT3286/pJYB288, *N*=17 cells). Trajectories were classified as a static species (red) and a fast diffusing dynamic species (blue). (**e**–**g**) Representative examples of static and dynamic trajectories are illustrated using the same colour code of **d**–**f** (red: static; blue: dynamic). Both panels depict one representative experiment of at least two biological replicates. (**h**–**j**) ParB_F_ and ParA_F_ choreography is concerted with HDR positions throughout the cell cycle. 2D localization density of (**h**) HDRs in *E. coli*, (**i**) ParB-mVenus and (**j**) ParA_F_-mVenus as a function of the cell cycle. Dashed purple lines in **i**,**j** provide a guide to the eye for the localization pattern of HDR maxima from **h**. Colour-coded scale (a.u.) represents HDR/ParB_F_/ParA_F_ density. *N* depicts the number of nucleoids analysed. All panels depict one representative experiment of at least five biological replicates.

Using two-colour 3D-SIM, we simultaneously imaged the nucleoid using HU-mCherry labelling and ParB_F_-mVenus. Volumetric images of the nucleoid revealed that HDRs are also a common feature of *E. coli* chromosomes (Fig. 4a and Supplementary Movie 10). HDR positions in *E. coli* were detected as previously described^26^ (see Methods for details). Interestingly, HDRs and ParB_F_ displayed a degree of colocalization (63±36%, *N*=4,744) larger than random (20%, Fig. 4b and Supplementary Figs 6A and 7A), suggesting that partition complexes may interact with high-density chromosomal DNA regions during segregation and persist throughout the cell cycle (Supplementary Fig. 7C). Next, we simultaneously imaged ParA_F_-GFP and HU-labelled nucleoids at super resolution. Interestingly, overlays of HDRs with ParA_F_ exhibited a large degree of positional correlation, even higher than that observed for partition complexes (82±24%, *N*=9,553; Fig. 4c and Supplementary Figs 6B and 7B). ParA and ParB*S* form dynamic complexes; thus, slight differences in position between HDRs/ParA-ParB*S* may arise from movement of complexes between acquisitions (∼20 s).

To investigate whether localization of ParA_F_ to HDRs influenced the process of segregation, we followed the dynamic movement of single ParA_F_ molecules in live cells using single-particle tracking PALM (spt-PALM)^33^,^34^. Wild-type and ParA_F_ mutants were labelled with the photo-activatable fluorescent protein mEos2. Histograms of the mean-squared displacement from individual trajectories of wild-type ParA_F_ revealed two distinct species: a dynamic population that explored large regions of the nucleoid and a static species that localized to discrete patches (Fig. 4d,e). The diffusion coefficients of these species were consistent with previous observations of DNA-associated proteins and free proteins^35^. The number of static ParA patches was similar to the number of ParA patches colocalizing with HDRs observed by 3D-SIM (compare panels c and e of Fig. 4). Strikingly, for the ParA mutant with reduced ns-DNA-binding capacity (ParA_F_^K340A^), the static population became negligible and the localization of ParA was homogeneous over the entire nucleoid (Fig. 4f,g). Importantly, this ParA mutation leads to abnormal positioning of partition complexes and to segregation defects (Fig. 3g–i), indicating ParA localization to HDRs is important for the volumetric localization and proper segregation of partition complexes.

Our results show that there is an apparent correlation between the 3D positions of HDRs and ParA/ParB*S* complexes. To determine whether this positional correlation is conserved during the cell cycle, we analysed the localizations of ParA and ParB*S* complexes together with those of HDRs as a function of nucleoid size, which directly correlate with the cell cycle stage^36^. First, we computed the probability density of localization of HDR as a function of nucleoid length and longitudinal position. Maxima in the HDR probability density represent regions where HDRs display a conserved or enriched subcellular localization (Fig. 4h). HDR maxima were localized at midcell for nascent nucleoids, and moved to quarter positions where they remained until the next cycle of cell division. By two-colour 3D-SIM, we simultaneously localized the positions of ParB_F_ complexes and calculated their probability density of localization as a function of cell cycle progression. Strikingly, we observed that the segregation pattern of ParB*S* complexes followed closely that of HDR maxima (Fig. 4i, see overlayed dashed line for density of HDR maxima), consistent with our previous studies of *B. subtilis* ParB^26^. Both HDR and ParB*S* maxima in the localization density maps forked at a nucleoid size of ∼2,300 nm (Fig. 4h,i, horizontal grey lines). Finally, we performed a similar experiment to detect the mean cell cycle positioning of ParA_F_ (Fig. 4j). ParA_F_ localized predominantly to the edges of the nucleoid for the majority of the cell cycle, consistent with the polar localization of ParA_F_ observed at fixed nucleoid sizes (Fig. 2c). In the localization density map, ParA_F_ maxima forked earlier than HDRs/ParB*S* (∼1,800 nm, horizontal grey line in Fig. 4j) at a nucleoid size matching the dwindling of chromosomal DNA density at the centre of the nucleoid (Fig. 4h). These findings are not inconsistent with the colocalization between HDRs and ParA patches at the single cell level, as kymograph maxima highlight a preferential positioning of HDRs/Par proteins within the nucleoid volume in a population. Interestingly, perturbation of HDRs using rifampicin treatment, an antibiotic known to block transcription and to affect chromosome structure, led to a spreading of HDRs positioning throughout the nucleoid volume as well as the cell cycle, and affected the positioning of both ParA and ParB in a very similar manner as that of HDRs (Supplementary Fig. 8).

## Discussion

In this study, we investigated the mechanism of segregation of two model partition systems in bacteria by combining 3D-SIM, MFM, spt-PALM and widefield deconvolution microscopy with quantitative high-throughput analysis. Our experiments demonstrate that all components of the partition machinery are localized within the nucleoid interior during segregation, and that the DNA-binding and ATPase activity of the ParA transport protein are essential for this localization. These results call for a re-evaluation of several aspects of previously proposed models.

Two main mechanisms were proposed to account for the segregation of bacterial partition complexes: filament and brownian-ratchet-like models^37^ (Fig. 1a). Filament models proposed that ParA functions as a nucleoid-spanning filament that either pushes or pulls partition complexes^19^,^20^. In contrast, we failed to observe extended ParA filaments using either 3D-SIM or spt-PALM. These results are compatible with recent observations in which it was shown by 2D sub-diffraction imaging that *Caulobacter crescentus* ParA does not form continuous filaments^25^. Overall, these results are inconsistent with extended ParA filaments or helical structures driving segregation of partition complexes^1^. Importantly, our ability to detect the 3D localization of ParA within the nucleoid allowed us to determine that ParA localizes specifically within the volume of the nucleoid. This volumetric localization of ParA is consistent with previous reports in other model systems^20^,^29^, suggesting that it may constitute a universal property of partition complexes. In addition, we observed by both 3D-SIM and spt-PALM that ParA forms small patches extending between nucleoid poles through the interior of the nucleoid (see model below).

On the basis of the observation of 2D diffusion of partition complexes on DNA-coated surfaces *in vitro*, most recent models postulated that partition complexes are segregated over the nucleoid surface via interactions with ns-DNA-bound ParA dimers or oligomers^21^,^22^,^23^,^24^. This model attributed a function to the cytosolic space between nucleoid and cell membrane because the high spatial confinement at this interface promotes frequent associations between plasmid-bound ParB and nucleoid-bound ParA^21^. It is worth noting, however, that general Brownian-ratchet-like mechanisms do not make specific statements about the spatial distribution of partition components. A variation of this model (DNA relay) proposed that, in addition to the brownian-ratchet, chromosomal DNA dynamics may also power partition complex propagation over the nucleoid surface^25^. These conclusions were mainly reached by simulations of diffusion of partition complexes in 2D. Here we show by several microscopy methods and for plasmid and chromosomal model systems that *in vivo* both partition complexes and the ParA ATPase localize predominantly within the nucleoid volume and in average to the longitudinal cell axis. Our findings are consistent with previous observations of the localization of *B. subtilis* ParB^26^, and are incompatible with partition complex segregation via surface-activated mechanisms requiring an enrichment of ParB*S* and ParA at the interface between nucleoid and cell membrane^24^,^25^.

Interestingly, our studies also reveal that the volumetric localization of ParA patches and partition complexes is highly correlated with the positions of HDRs. HDRs are present in both *B. subtilis*^26^ and *E. coli* (Fig. 4) and represent regions where DNA is more highly compacted, probably because of the stochastic, dynamic association of chromosomal interaction domains^26^. The localization pattern of ParB_F_ maxima seems to closely follow that of HDR maxima throughout the cell cycle (Fig. 4h,i), and even upon perturbation of the nucleoid structure ParA and ParB mirror the altered localization of HDRs (Supplementary Fig. 8), consistent with ParB*S* complexes being constitutively located close to regions of high chromosomal DNA density. Remarkably, segregation patterns of HDR maxima and ParB are also highly correlated in *B. subtilis*^26^, despite the different cell cycle localization patterns of ParB in these species (Supplementary Fig. 9). However, in *B. subtilis,* ParB patterns do not follow HDR maxima as closely as those reported in *E. coli,* which could be explained by important differences in the genomic and spatial sizes of partition complexes of Par systems from these two species. In *E. coli*, replication origins are located at the centre of new cells and segregate to quarter positions concomitantly with replication^38^,^39^,^40^,^41^,^42^. This precise choreography closely resembles that of HDR maxima, and suggests that they may correspond to replication origin regions as in *B. subtilis*^26^. Overall, these observations suggest that the subcellular localization of partition complexes may also be influenced by the host replication/segregation machinery.

In *E. coli*, HDRs occupy the nucleoid volume and are more likely found close to the longitudinal nucleoid axis (Fig. 4a–c and Supplementary Fig. 9)^26^. ATP-bound ParA interacts with ns-DNA and with itself^43^,^44^. Thus, these two activities and the volumetric localization of HDRs are consistent with the observation that ParA forms patches localizing at a discrete number of positions within the nucleoid volume. This may serve for the spatial concentration of the cellular pool of ParA into a small number of locations distributed inside the nucleoid, as suggested by the high degree of ParA_F_ colocalization with HDRs (>80%; Fig. 4c and Supplementary Fig. 7B) and the absence of ParA patches when its nonspecific interaction with DNA is impaired (Fig. 4f,g). In contrast to ParB_F_, ParA_F_ maxima are located at the edges of the nucleoid, and split when chromosomal DNA density at the centre of the nucleoid begins to dwindle (Fig. 4j), consistent with the oscillatory behaviour of ParA_F_ (ref. 45). Thus, the segregation pattern of ParA maxima seems in part to define the subcellular localization of ParB*S* complexes, and conversely ParA appears depleted in regions of high ParB*S* density. These observations are consistent with partition complexes dynamically following ParA concentration gradients^29^, triggering ParA dissociation by direct interaction with ParB^22^.

In absence of ParA_F_, partition complexes displayed a very dynamic behaviour and lost their volumetric localization pattern. These results indicate that ParA directly participates in the tethering of partition complexes to the nucleoid interior. Importantly, the ability of ParA_F_ to bind ns-DNA and to hydrolyse ATP through ParB_F_ stimulation were the key for the maintenance of partition complexes within the nucleoid volume (Fig. 3). In these mutants, partition complexes were excluded from the nuclear volume, likely because of impaired formation of ParA concentration gradients^46^ and localized to the space between nucleoid and cell membrane mostly at cell poles. These findings are in good agreement with previous observations of higher intracellular mobility of *par* minus plasmids^47^ and with nucleotide-bound state of ParA and ns-DNA binding playing a role in the longitudinal positioning of partition complexes^1^.

**Figure 5:**
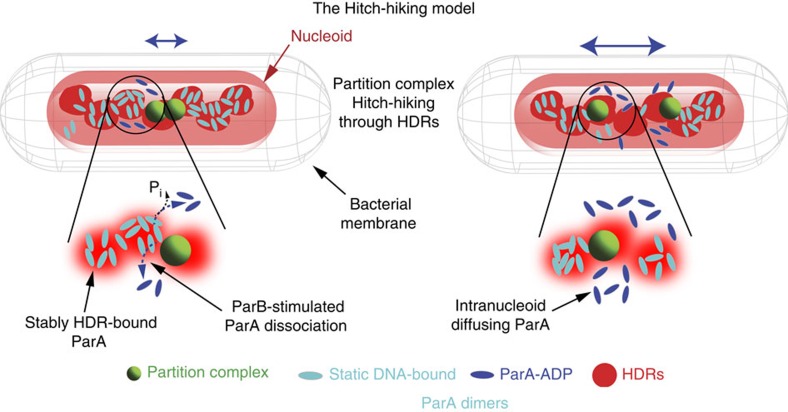
Hitch-hiking model for bacterial chromosome segregation. The Hitch-hiking model for bacterial chromosome segregation. See text for description. Cell outline is shown as a grey mesh, nucleoid as a red cylinder, ParB*S* partition complexes in green, static DNA-bound ParA in cyan, free ParA-ADP in blue and HDRs as red diffuse circles.

Thus, we favour a mechanism (‘hitch-hiking' model) in which partition complexes are segregated by being recruited to high-density regions within the nucleoid by interactions mediated by the ParA ATPase. In this model (Fig. 5),

1. ParA assembles in small patches at regions of high DNA density. This could arise by the ns-DNA-binding activity of ParA and the local high concentration of DNA within HDRs. Dissociation of ParA from ns-DNA requires either ATP hydrolysis^44^ or direct interaction with ParB^22^; thus, dissociation of short ParA oligomers is prevented when ParA is at HDRs. Without the presence in the close proximity of ParB or partition complexes, these ParA oligomers are relatively stable.
2. Partition complexes are most likely formed by nucleation and caging of hundreds of ParB dimers around a cluster of *parS* sites into small complexes (<150 nm, Supplementary Fig. 2)^13^. ParB and *parS*-bound ParB strongly stimulate the ATPase activity of ns-DNA-bound ParA complexes (40- and 120 fold, respectively^18^), leading to their dissociation from DNA^24^,^37^. Thus, interactions of ParB with HDR-bound ParA patches may trigger their progressive dissociation from HDRs, and subsequently release partition complexes from HDRs (Fig. 5, left panel). Unbound/ADP-bound ParA dimers can then rapidly diffuse, reload ATP to become competent for ns-DNA binding (after a time delay^24^) and preferentially oligomerize at new HDRs (Fig. 5, right panel).
3. This ‘scanning' allows ParA oligomers to be up to date with changes in the conformation of the nucleoid, and to hitch-hike on HDRs.
4. Interactions of ParB with ns-DNA-bound ParA are dynamic and could lead to a bias in the diffusion of partition complexes towards HDR-bound ParA patches. The radial concentration of ParA from the cytoplasm towards the longitudinal axis of the nucleoid, where DNA/ParA is more concentrated, could drive the partition complex within the nucleoid volume, while local gradients in ParA along the longitudinal axis of the nucleoid temporarily hold it in place. Once ParB has depleted local ParA in its vicinity, it can diffuse to the most proximal HDR enriched in ParA, hence moving along the heterogeneous distribution of ParA. The confinement diameter of ∼200 nm of ParB foci obtained from tracking experiments indicates that the area explored by partition complexes is comparable to the distance between ParA patches (248±74 nm, see Supplementary Fig. 10). Thus, diffusion should allow partition complexes to bridge two ParA patches without stalling. In brief, partition complex movement is composed of a brownian diffusion component and a directional bias, the essential constituents of a brownian-ratchet (Fig. 5).

Recently, we observed that the *B. subtilis* chromosome dynamically condenses/de-condenses during its replication cycle. Decondensation of the origin domain (containing most chromosomal *parS* sites) seems to occur concomitantly with replication initiation and with the relocalization of the origin from nucleoid pole to the middle of the new nucleoid. This relocalization is accompanied by a global change in replichore organization from a longitudinal to a transverse orientation^26^,^27^. Interestingly, a similar change in replichore organization was recently proposed for *E. coli*^42^. Thus, origin decondensation upon replication may lead to the preferential relocalization of ParA at HDRs other than the origin. We hypothesize that newly replicated origin regions may thus follow the bias in ParA localization to move away towards their new subcellular localizations. Future experiments will need to be performed to test these hypotheses.

Members of the ParA/MinD family are involved in the subcellular positioning of a large number of protein machineries with a diverse variety of functions, including the conjugative transfer and type IV pili apparati, cellulose synthesis devices and cytoplasmic chemotaxis clusters^2^,^48^. Presumably, because of the nucleoid acting as a diffusion barrier and/or because of nucleoid exclusion forces, such large complexes have been assumed to occupy the cytosolic space or to use the inner bacterial membrane as a scaffold. Our findings reinforce the need of volumetric super-resolution imaging of subcellular structures and machineries on the cellular context to determine the role of subcellular ultrastructures, such as the nucleoid or the cell membrane, in the process of transport. It remains to be demonstrated whether, as it occurs for the F-plasmid and *B. subtilis* chromosome, such transport systems employ the scaffold of the nucleoid and may thus co-opt other cellular machineries for the intracellular transport of large cargoes. We speculate that volumetric patterning of ParAs may have been evolutionarily advantageous in bacteria and archaea for a variety of biological functions to serve as molecular beacons for positioning and/or segregating all kinds of cargoes.

## Methods

### Bacterial strain and plasmids and growth conditions

Strains, plasmids and growth conditions are described in Supplementary Methods. For microscopy and plasmid stability assays, cultures were grown at 30 °C with aeration in a supplemented M9 minimal medium (MGC; Supplementary Methods). A full list of strains employed in this work is given in Supplementary Methods.

### Fluorescence microscopy

3D-SIM imaging was performed on an OMX V3 microscope (Applied Precision), using 405, 488 and 568 nm laser lines to excite DAPI, mVenus and mCherry respectively^26^,^49^. To perform dual colour volumetric imaging, 15-17 slices were acquired sequentially for each colour with a Z step of 125 nm between each slice. Reconstruction and alignment of 3D-SIM images was performed using softWoRx v5.0 (Applied Precision). Widefield deconvolution imaging was conducted on the same experimental set-up and used the same channel alignment procedure and algorithm. Refer to Supplementary Methods for more details.

Single-particle tracking PALM and MFM were conducted on home-built experimental set-ups based on Zeiss Axiovert 200 microscopes. Volumetric acquisitions for MFM imaging were permitted by inserting custom optical elements into the emission pathway between the microscope tube lens and the EM-CCD camera (DU-897; Andor). More details on the experimental set-ups can be found in the Supplementary Methods.

### Data analysis

Data analysis was performed as described in Supplementary Methods and elsewhere^26^. Briefly, cells and nucleoids were automatically segmented using a 3D maximum entropy thresholding algorithm for binarization. The same methods were used to detect minimum and maximum surface/volume values for cells and nucleoids, respectively. Analysis of nucleoid length, width and height used the FWHM of the nucleoid long and short axes. Nucleoid dimensions were then used to draw the nucleoid contour in density histogram representations. ParB foci in each cell were detected as 3D local maxima, using a multidirectional derivation of the intensity scalar vector, and their positions were normalized by the nucleoid dimensions. Nucleoids depicted in figures were segmented by visual impression for 3D rendering of nucleoid surfaces (Figs 1d,e, 2a,b, 3b,e,h and 4a). 2D histograms of the positions of the ParB foci in the nucleoid were constructed with nucleoids of length <1,500 nm. The selected nucleoids were resampled 100 times using a bootstrapping method, and foci position orientations were randomized along the long, short and vertical axes of the nucleoids before computing 2D histograms. 2D histograms were generated by computing the 2D spatial distribution of number of local maxima weighted by their corresponding local voxel intensity in the 3D-SIM stack. The mean histogram was then calculated from the 100 smoothed histograms built from the 100 bootstrapped samples. Note that for YZ histogram construction, solely ParB foci whose longitudinal coordinates lie within 60% of the nucleoid length were considered in order to avoid the contribution from ParB foci at the nucleoid poles. Histograms of ParA distribution within the nucleoid were computed the same way but ParA foci/patch orientation along the nucleoid long axis was set to keep the brightest patches on one side. spt-PALM data analysis is described in Supplementary Methods.

### Data availability

The data that support the findings of this study are available from the corresponding author upon request.

## Additional information

**How to cite this article:** Le Gall, A. *et al*. Bacterial partition complexes segregate within the volume of the nucleoid. *Nat. Commun.* 7:12107 doi: 10.1038/ncomms12107 (2016).

## Supplementary Material

Supplementary Information Supplementary Figures 1-10, Supplementary Table 1, Supplementary Methods and Supplementary References

Supplementary Movie 1 3D-SIM of an E. coli nucleoid (solid red) carrying a wild type mini-F plasmid with parBF tagged mVenus (green). Strain DLT3053/pJYB234.

Supplementary Movie 2 3D volume rotation of a B. subtilis nucleoid (solid red) with parBBsu tagged GFP (green) imaged in 3D-SIM. Strain HM671.

Supplementary Movie 3 3D volume of ParAF (blue) with side projections of ParAF densities, imaged in 3D-SIM. Strain DLT3053/pJYB243.

Supplementary Movie 4 3D volume of an E. coli nucleoid (solid red) ParAF (blue) imaged in 3D-SIM. Strain DLT3053/pJYB243.

Supplementary Movie 5 3D epifluorescence imaging of E. coli cells harboring F-plasmids lacking ParAF and with ParB tagged with mVenus. Strain DLT3053/pJYB263.

Supplementary Movie 6 3D epifluorescence imaging of E. coli cells harboring F-plasmids lacking ParAF and with ParB tagged with mVenus. Strain DLT3053/pJYB263.

Supplementary Movie 7 3D epifluorescence imaging of E. coli cells harboring F-plasmids with ParAF-K120Q, an allele in which the ParBF stimulation of ParAF's ATP hydrolysis is abolished, and with ParB tagged with mVenus. Strain DLT3053/pJYB277.

Supplementary Movie 8 3D epifluorescence imaging of E. coli cells harboring F-plasmids with ParAF-K340A, an allele deficient in ns-DNA binding, and with ParB tagged with mVenus. Strain DLT3053/pJYB279.

Supplementary Movie 9 3D epifluorescence imaging of E. coli cells harboring F-plasmids with ParAF-K340A, an allele deficient in ns-DNA binding, and with ParB tagged with mVenus. Strain DLT3053/pJYB279.

Supplementary Movie 10 3D-SIM of an E. coli nucleoid (solid red). Strain DLT3053/pJYB234.
