# Supplementary material for: Bacterial partition complexes segregate within the volume of the nucleoid
Source: Nat Commun. 2016 Jul 5;7:12107. doi: 10.1038/ncomms12107 (PMC4935973; doi:10.1038/ncomms12107)
Supplement: Supplementary Information — Supplementary Figures 1-10, Supplementary Table 1, Supplementary Methods and Supplementary References [file ncomms12107-s1.pdf]

I. Supplementary figures

Supplementary Figure 1

Nucleoid dimensions and number of partition complexes per nucleoid

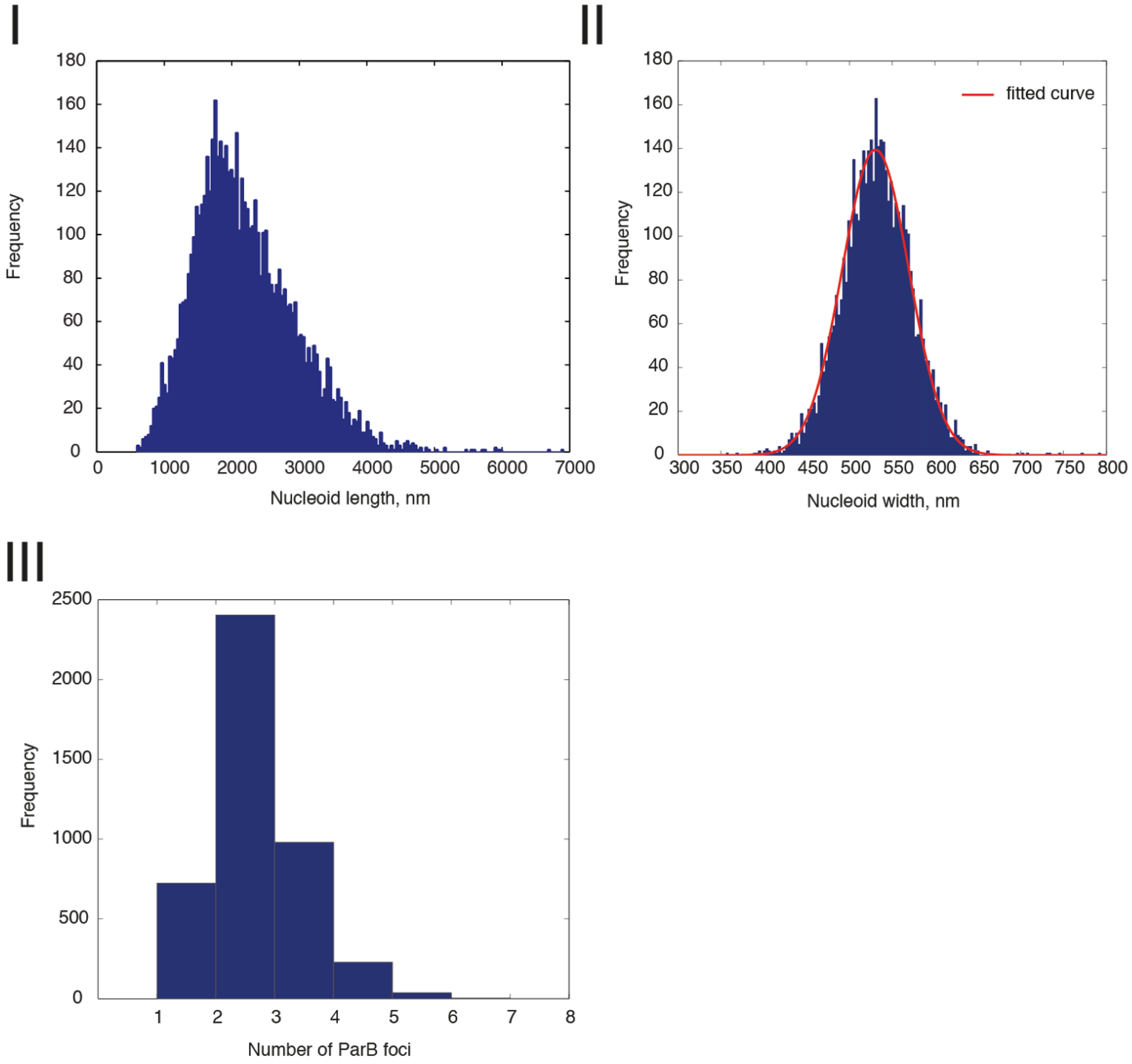

Supplementary Figure 1: Distribution of nucleoids (I) lengths (full width half maximum) and (II) widths for nucleoids of strain DLT3053/pJYB234 labelled with HU-mCherry (N = 4744 nucleoids). Fitting of the nucleoid width distribution with a Gaussian (red curve) yielded a mean width of 530 ± 50 nm (mean ± standard deviation). (III) Distribution of the number of partition complexes per nucleoid. The mean number of partition complexes per nucleoid is 2.2 ± 0.8.

Supplementary Figure 2

Partition complex size

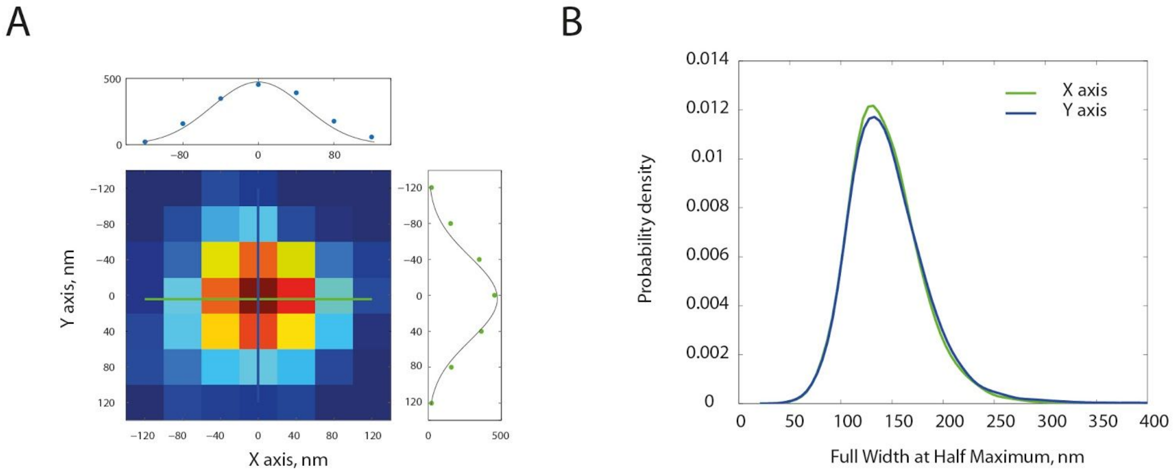

Supplementary Figure 2: (A) Single $ParB_F$ focus imaged in 3D-SIM. 2D Gaussian fit yielded a Full Width at Half Maximum (FWHM) of 130 and 124 nm on X and Y directions respectively (green and blue lines). (B) Kernell density distributions of the FWHM of 7364 $ParB_F$ foci obtained from gaussian fits. The median ± standard deviation of the width along X and Y directions is 139±37 and 141±41 nm, respectively. These measurements are slightly higher than the 3D-SIM resolution (~120nm) and are consistent with previous studies showing the confinement zone of ParB foci in live cells are in the scale of 150nm [1].

Supplementary Figure 3

Partition complex localization inside the nucleoid

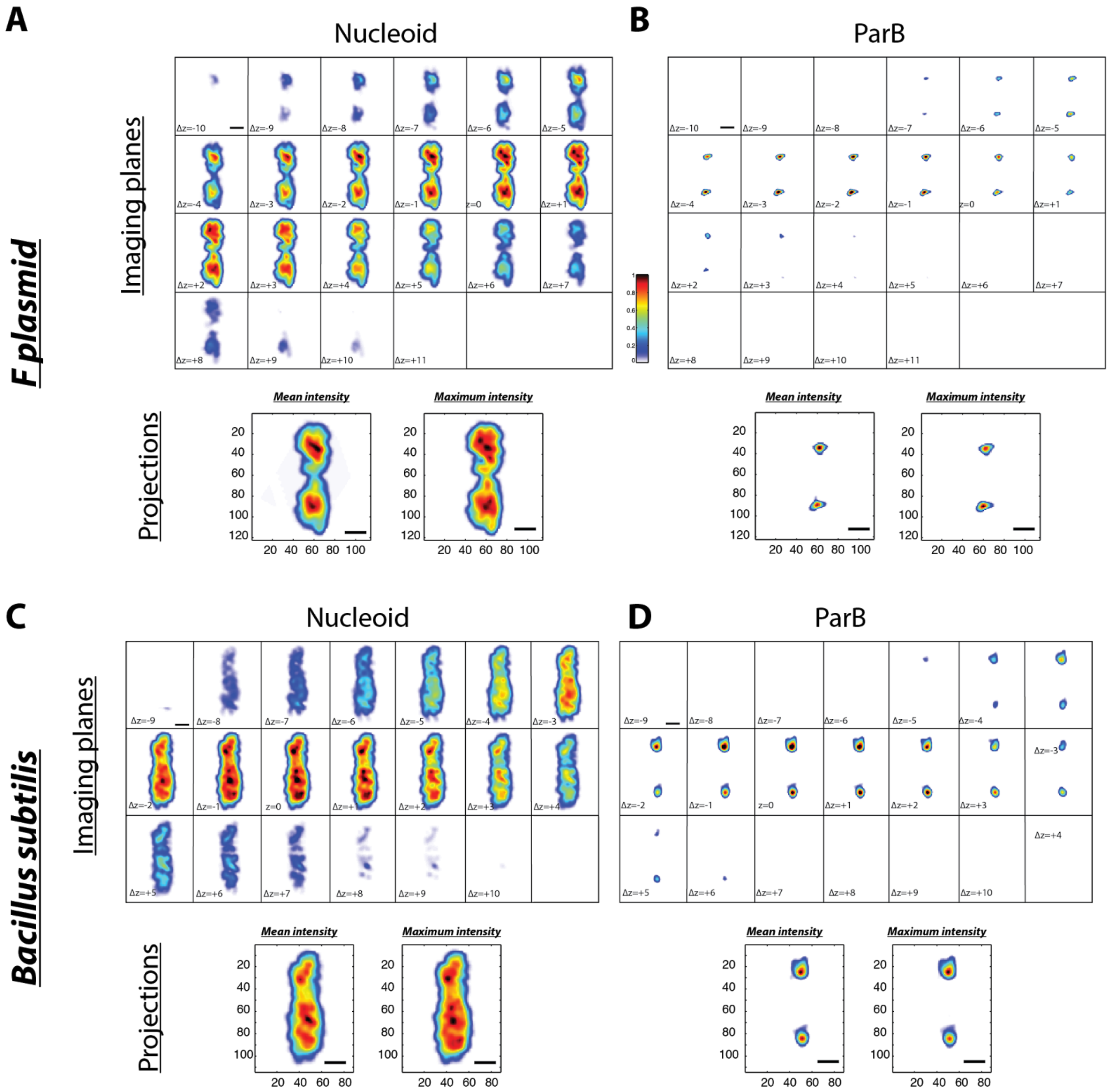

Supplementary Figure 3: x-y sections and z projections nucleoids and ParB 3D-SIM reconstructions for (A-B) F plasmid (ParB-mVenus and HU-mCherry labelled nucleoid) and (C-D) *B. subtilis* (ParB-GFP and DAPI stained nucleoid) from panels D and E of Figure 1 from the main text. Top panels represent the Z-planes of the nucleoid (A-C) and ParB (B-D). The spacing between each Z-plane (ΔZ) corresponds to 125 nm. Lower panels represent the maximum and mean intensity projections for the nucleoid and ParB in (A-C) and (B-D) respectively. Scale bar is 400 nm.

Supplementary Figure 4

Multi Focus Microscopy raw data

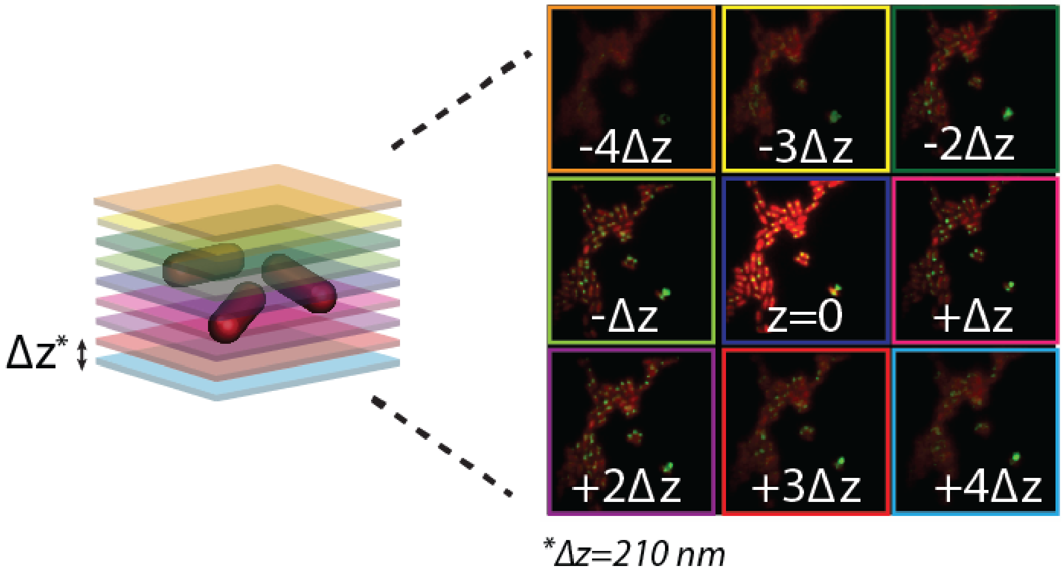

Supplementary Figure 4: Instantaneous 3D multi-focus microscopy (MFM) raw experimental data of $ParB_F$ and nucleoids (Strain DLT3053/pJYB234). Left: MFM scheme for fast volumetric acquisitions representing the acquired nine Z-planes imaged across bacteria. Right: corresponding Z-planes represented as acquired on the camera chip without optical aberrations correction. ΔZ* represents the mean distance between planes (independent of color). The distance between planes varies with wavelength, but this variation is less than 5-10% between the emission channels used here.

Supplementary Figure 5

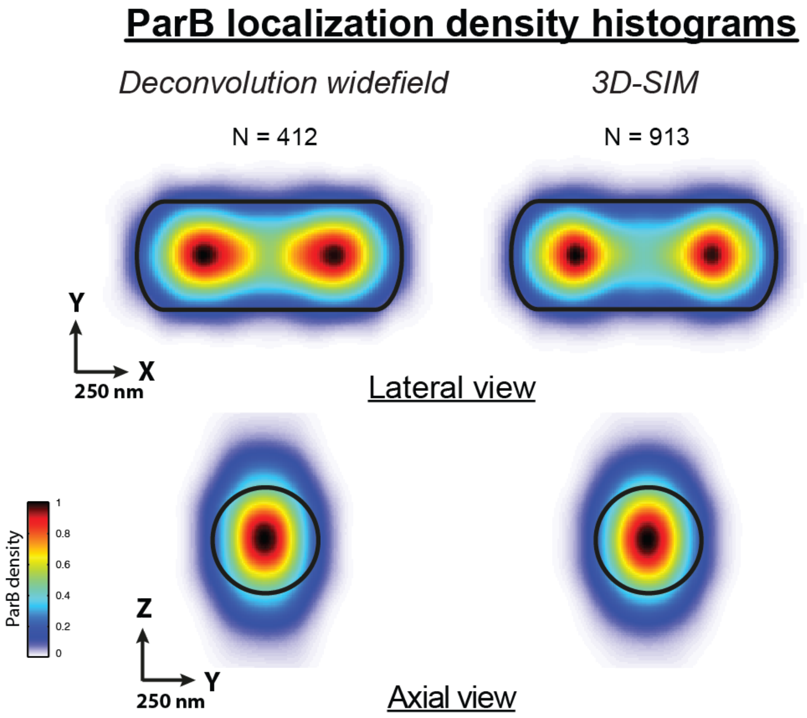

Supplementary Figure 5: Localization of partition complexes is equivalent when imaged by 3D-SIM or wide field deconvolution microscopy. Two-dimensional density distributions of $ParB_F$ intensity signal along the XY (lateral) and YZ (axial) planes constructed from deconvolution widefield and 3D-SIM acquisitions, respectively (strain DLT3053/pJYB234). Only nucleoids of similar width and length below 1500 nm were used for calculation of $ParB_F$ densities (see Methods for details). No significant differences can be observed between the two acquisitions modalities. Dimensions of nucleoid contours (solid black lines) were measured as the full width at half maximum of the intensity signal along each nucleoid dimension. *N*: number of nucleoids analyzed for each histogram.

Supplementary Figure 6

ParB$_F$-*parS* complexes and ParA$_F$ show a high degree of colocalization with DNA dense regions (HDRs).

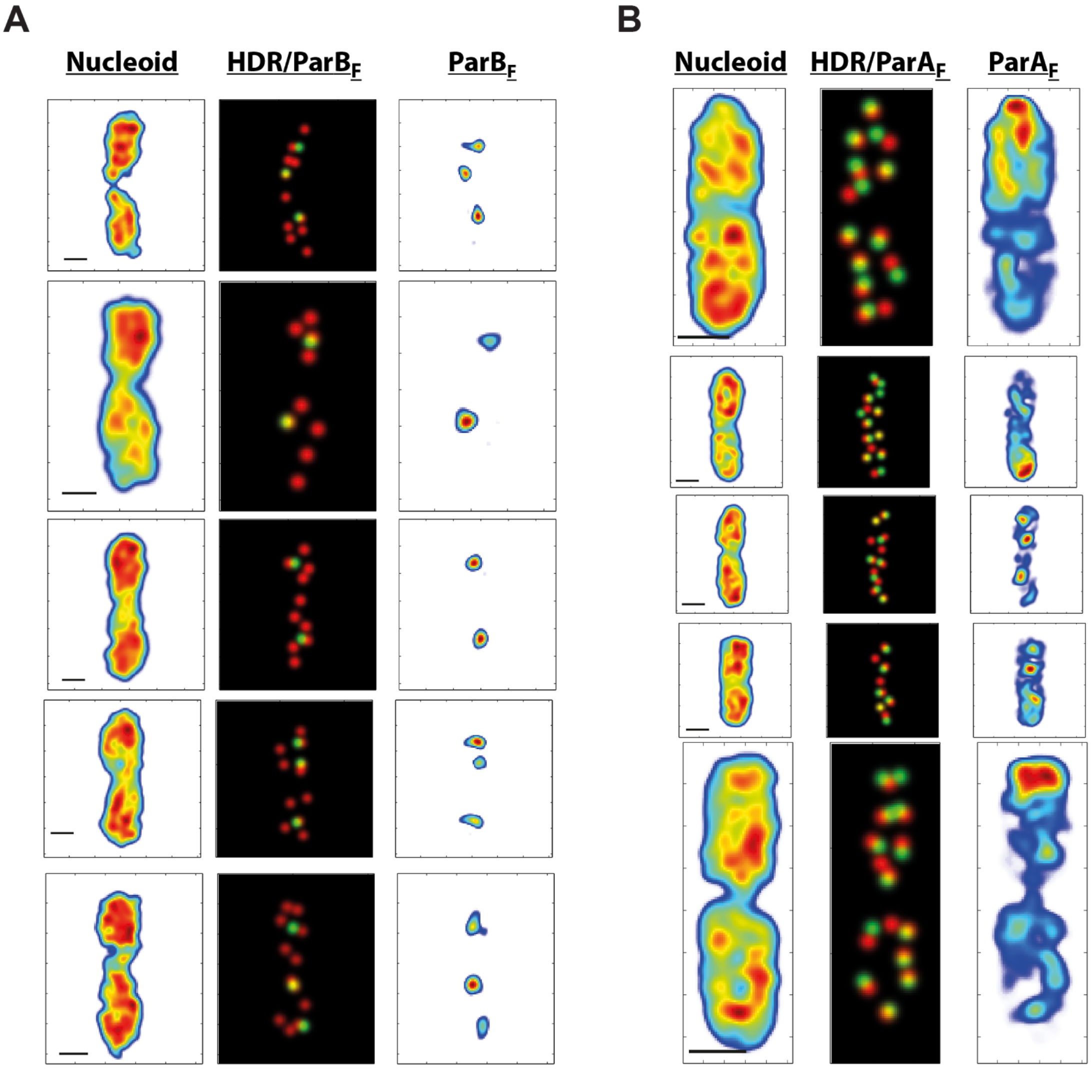

Supplementary Figure 6: **(A)** Left: maximum intensity projections (MIP) of individual nucleoid tagged with HU-mCherry (Strain DLT3053/pJYB234). Small spatial features of nucleoid volumes were reconstructed and highlighted by combining in the Fourier space low with high spatial frequencies multiplied by an enhancement factor (typically ~1). Right : MIP of the same nucleoids but for the channel corresponding to the ParB-mVenus signal. Middle : Top projection of HDRs identified through local maxima detection in each nucleoid and represented in 2D as red Gaussians while ParB foci are represented as green Gaussians. HDRs that colocalize with ParB positions show up as overlapping Gaussians and appear in yellow. Scale bar is 400 nm.

(B) Same representations as in (A) but in a cell line where ParA$_F$ is tagged with mVenus (strain DLT3053/pJYB243). Note that due to nucleoids dynamics between the successive acquisitions and our temporal resolution, most HDRs, ParB$_F$ and ParA$_F$ positions will slightly move but a strong correlation between their positions can still be noted.

Supplementary Figure 7

Measured distributions of 3D distances between $ParB_F$-*parS* complexes or $ParA_F$ to the most proximal HDR.

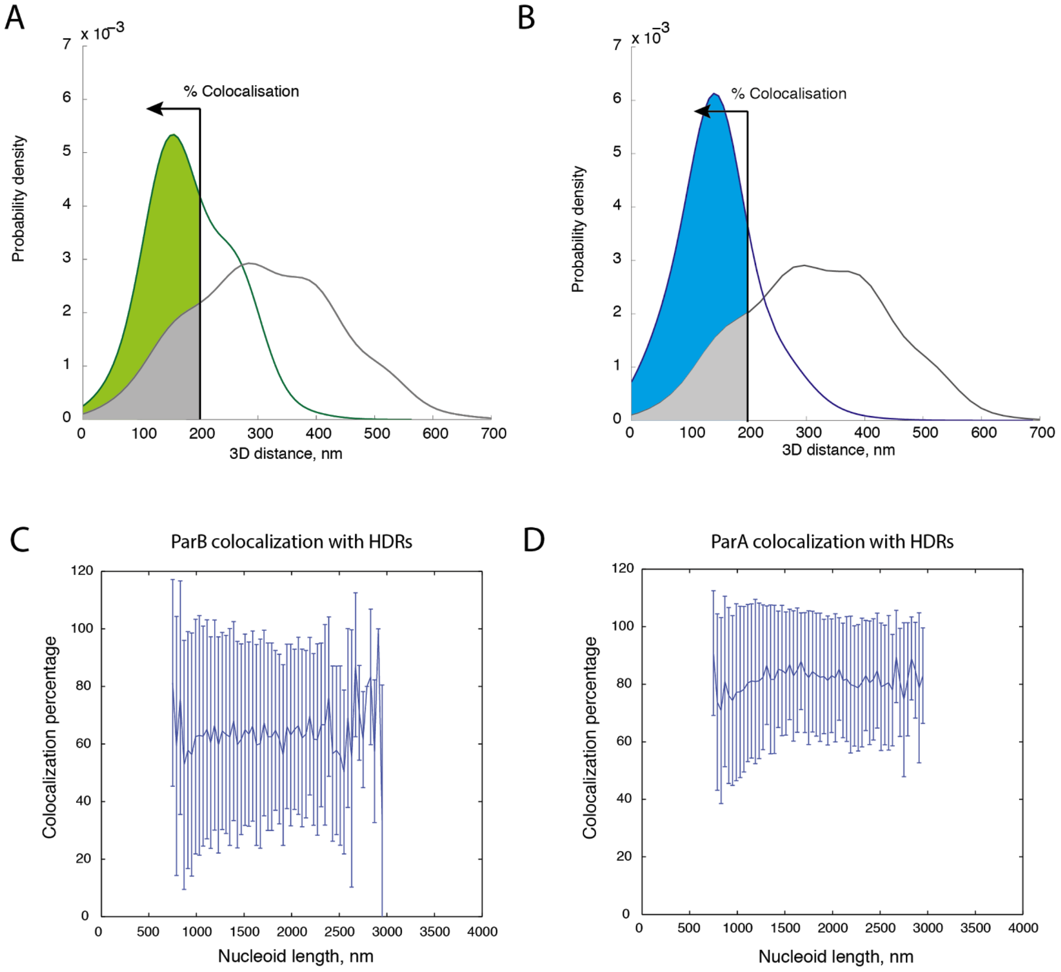

Supplementary Figure 7: **(A)** Kernel density estimation of the histograms of 3D distances for ParB foci (green line) and simulated loci with random positions (grey line) in which the localization probability is homogeneous within the entire nucleoid volume (strain DLT3053/pJYB234). Percentages of co-localization were calculated by normalizing the number of 3D distances below 200 nm (chose as a compromise between the 3D-SIM lateral and axial resolutions) by the total number of measured distances. $ParB_F$ colocalization yielded 63 ± 36 % (mean ± standard deviation of the mean colocalization percentage among cells) and the negative control 20 ± 29 % measured on 4744 nucleoids.

(B) Same distributions as in (A) but in a cell line where $ParA_F$ was tagged with mVenus (strain DLT3053/pJYB243). $ParA_F$ colocalization to HDRs yielded 82 ± 24% and the negative control 19 ± 22 % measured on 9553 nucleoids.

(C-D) Percentage of colocalization of ParA (C) and ParB (D) with HDRs as a function of cell cycle (nucleoid length). The mean colocalization percentage of Par proteins with HDRs was computed for each cell and averaged for nucleoids of similar length. Error bars represent the standard deviation among nucleoids of the same length.

Supplementary Figure 8

HDRs and Par proteins positioning in wild type and rifampicin treated cells

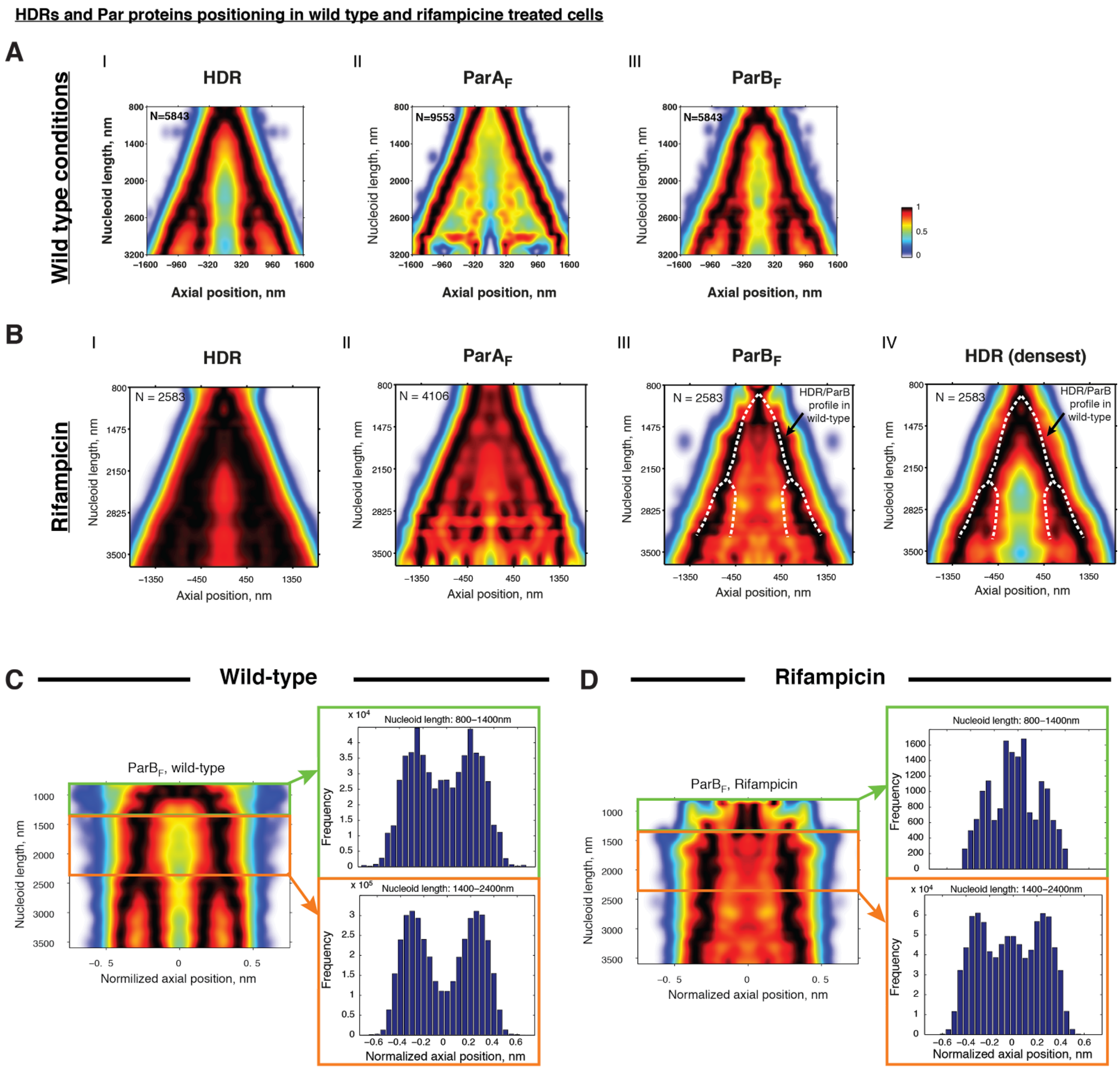

Supplementary Figure 8: (**A**) Panels I-II-III are reproduced from panels D-F-E of Figure 4 of the main text for visual comparison with panel (B). **(B)** perturbed HDRs (I), $ParA_F$ (II) and $ParB_F$ (III) positioning upon rifampin treatment (200µg/ml for 3h30). **(C-D)** Left panels show the normalized axial localization densities of ParB-mVenus as a function of nucleoid length for wild-type (C) and rifampicin treated cells (D). Right panels display histograms of the normalized axial localization of ParB-mVenus for newborn nucleoids (800 < nucleoid length < 1400 nm, green frame) and longer nucleoids (1400 < nucleoid length < 2400 nm, orange frame). Rifampicin treatment is known to block transcription initiation which affects chromosome structure while moderately affecting its organization [2,3]. Such treatment leads to a broadening in the distribution of HDRs positioning throughout the nucleoid volume as well as the cell cycle. These changes are also observed for both ParA and ParB, indicating that changes in the positions of HDRs lead to segregation defects (see dashed line in panels III and IV providing a guide to the eye

for the localization pattern of HDR/$ParB_F$ maxima from panels II-III in (A)). Maxima of ParB at the centre of the nucleoid in newborn cells and at roughly quarter positions in older cells mirrors the localization of the densest HDRs (panel IV). This suggests that the perturbed ParB localization pattern may still be guided by the localization of dense HDRs. Color-coded scale (a.u.) represents HDR/$ParB_F$/$ParA_F$ density. *N* depicts the number of nucleoids analyzed.

Supplementary Figure 9
***E. coli* and *B. subtilis* HDR density histograms**

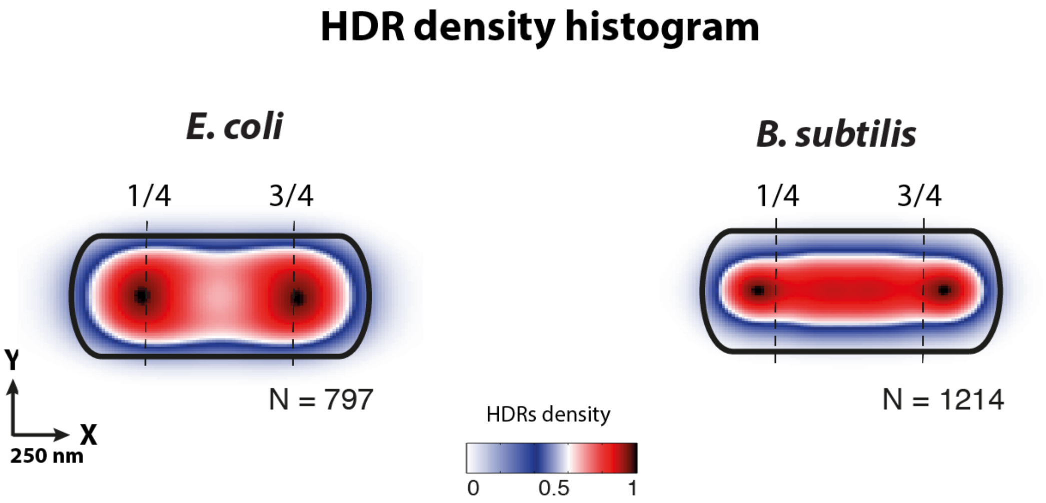

Supplementary Figure 9: Two-dimensional density distributions of HDR intensity signal along the XY (lateral) plane for *E. coli* (left, nucleoids labelled with HU-mCherry) and *B. subtilis* (right, nucleoids stained with DAPI) strains, DLT3053/pJYB234 and HM671, respectively. Only nucleoids of similar width and length below 1500 nm were used for calculation of HDR densities (see Methods for details). Nucleoid quarter positions are indicated on each histogram as vertical dashed lines to help visual comparison of the more polar enriched localization of HDRs in *B. subtilis* nucleoids as compared to *E. coli* nucleoids. Dimensions of nucleoid contours (solid black lines) were measured as the full width at half maximum of the intensity signal along each nucleoid dimension. N: number of nucleoids analyzed for each histogram. All panels of the figure represent one representative experiment of at least five biological replicates.

Supplementary Figure 10

Diffusion allows partition complexes to bridge two ParA patches without stalling

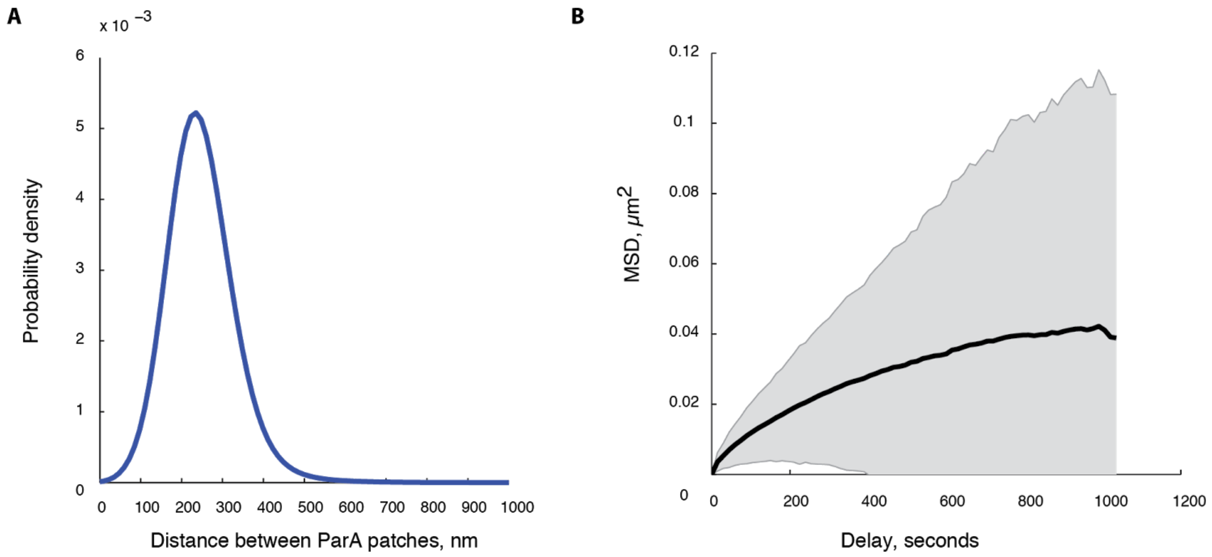

Supplementary Figure 10: (**A**) Distribution of nearest-neighbour distances between ParA patches (248 ± 74 nm, mean ± std). N = 9553 nucleoids analysed. (**B**) Averaged mean squared displacement from 758 $ParB_F$ foci trajectories (dark solid line) and standard deviation (grey shadow area). ParB-mVenus was tracked for 18 min every 15 s. The average MSD reaches a plateau, indicating that most partition complexes exhibit a confined motion within the nucleoid. The confinement diameter of ~200 nm indicates that the area explored by ParB foci is comparable to the average distance between ParA patches.

II. Supplementary Table 1

Rate of mini-F plasmid loss in wild-type and mutants

**Plasmids**	**Characteristics**	**Loss rate in % of loss per cell per generation (±standard deviation)**	**Percentage of cells carrying the F-plasmid at time 0**
**pJYB234**	WT *parB*$_F$-mVenus	**0.06 % (±0.03)**	**99 %**
**pJYB263**	Δ*parA*$_F$ *parB*$_F$-mVenus	**4.34 % (±1.74)**	**82 %**
**pJYB277**	*parA*$_F$-K120Q *parB*$_F$-mVenus	**4.55 % {4.0%, 5.1 %}**	**63 %**
**pJYB279**	*parA*$_F$-K340A *parB*$_F$-mVenus	**4.95 % {5.3%, 4.6%}**	**68 %**

Supplementary Table 1: Plasmid loss rate measurements on *E.coli* strains carrying mini-F constructs. Assays were performed to screen for the appearance of cells lacking the mini-F construct over long-term population growth. The third column of the table lists the percentage of plasmid loss per generation in each strain (average from at least three experiments except for duplicates indicated in brackets) while the fourth column depicts the percentage of cells carrying the plasmid at the time of dilution from exponential growth in the presence of chloramphenicol to same media without chloramphenicol (t0).

III. Supplementary Methods.

Strains, media, and growth conditions

Nutrient agar (Euromedex) was used for routine selection and maintenance of bacteria. *B. subtilis* cells were grown at 30°C in M9 minimal media containing 0.5% glucose, 0.5% succinate and 0.1% glutamate for slow growth conditions while *E. coli* bacteria were cultivated in M9-glycerol (M9 minimal media supplemented with 0.4% glycerol, 0.2% casamino acids, 0.04mg.ml^{-1} thymine, 0.2mg.ml^{-1} leucine). For selective bacterial growth, the following concentrations of antibiotics were used (µg.ml^{-1}): chloramphenicol (Cm, 10), spectinomycin (Sp, 20) for *E. coli* strain, and Kanamycin (Cm, 1) for *B. subtilis* HM671 strain. *B. subtilis* HM671 strain was derived from the prototrophic strain PY79 [4]. *E. coli* DLT3053 was derived from DLT1215 [5] by P1 transduction of the *hu*-mCherry *frt::kan::frt* cassette (SS6282; [6]) to replace wild-type *hupA* on the chromosome for expression from the endogenous promoter. Mini-F plasmids, introduced by transformation in DLT1215 (pJYB229 and pJYB288) or DLT3053 (all others), are listed in the table below:

**Plasmids**	**Characteristics/Relevant properties**	**Reference**
pJYB229	mini-F wild type; parA$_F$-mEos2	this work
pJYB234	mini-F wild type; parB$_F$-mVenus	[7]
pJYB243	mini-F wild type; parA$_F$-mVenus	[1]
pJYB263	mini-F; ΔparA$_F$ parB$_F$-mVenus	this work
pJYB277	mini-F; parA$_F$-K120Q parB$_F$-mVenus	this work
pJYB279	mini-F; parA$_F$-K340A parB$_F$-mVenus	this work
pJYB281	mini-F; parA$_F$-K120Q-mVenus	this work
pJYB283	mini-F; parA$_F$-K340A-mVenus	this work
pJYB288	mini-F; parA$_F$-K340A-mEos2	this work

In vivo imaging

Before image acquisitions, microscopy coverslip and glass slides were rinsed with acetone and dried over an open flame to eliminate any remaining fluorescent contaminations. Next they were placed in a rack (to ensure complete cleaning from both sides of the slide) and plasma cleaned for 30 minutes using a Femto plasma cleaner (Diener electronics, Germany).

A frame of double-side adhesive tape was placed on a glass slide and a ~5mm channel was extruded from its center. 50 µl of 2 % melted agarose (diluted in M9 media, melted at 90°C) were spread on the center of the glass slide and covered with a second glass slide to ensure a flat agarose surface. The sandwiched slides were kept on a horizontal position for 5 min under external pressure at room temperature (RT) to allow the agarose to solidify. The high agarose concentration improves the stability of the pad during 3D-SIM and 3D-PALM experiments. The top slide was then carefully removed when bacteria were ready to be deposited in the agar pad (below).

Cells were harvested during the exponential phase (optical density at 600 nm: ~0.3). In rifampicin treatment experiments, the antibiotic was added at a concentration of 200µgl/ml and incubated for 3h30 prior to harvesting the cells. If necessary, cells were stained by adding, either simultaneously or individually, 7.5µl of a 0.3µM solution of DNA dye (DAPI, Invitrogen) to 1ml of bacterial suspension. After five minutes of incubation in the dark, the bacterial aliquot was spun down in a bench centrifuge at RT at 6973g for 1.5 minutes for *E. coli* and 1377g for 4 minutes for *B.subtilis* cell lines. The supernatant was then discarded and the pellet suspended in 20-30µl minimal media. 10 µl of the resulting bacterial solution were pipetted onto the agar.

After deposition of bacteria, the second face of the double side tape was removed and the pad was then sealed with a clean coverslip and kept in the dark for at least five minutes to allow for regular spreading of bacteria over the surface.

Mini-F stability assay

Fresh overnight cultures of DLT3053 strain containing a mini-F plasmid, grown in M9–glucose–casamino acid medium containing selective antibiotics (chloramphenicol), were diluted 400-fold into the same medium and regrown to A_{600}=0.25. Samples were then diluted serially into fresh M9 medium without the antibiotic selective for the plasmid under test and were processed as described previously [8].

Fluorescence Microscopy (3D-SIM, widefield deconvolution microscopy and MFM)

Cells from exponential cultures were transferred to a slide with a 2% agarose pad buffered with minimal medium (without CSA) and covered with a coverslip. 3D-SIM imaging was then performed on an OMX V3 microscope (Applied Precision) as previously described [9,10]. 405, 488 and 568 nm laser lines were used to excite DAPI, mVenus and mCherry respectively. Dual color volumetric images were acquired sequentially to avoid channels excitation and fluorescence crosstalks. Reconstruction and alignment of 3D-SIM images was performed using softWoRx v5.0 (Applied Precision). Channel specific Optical Transfer Functions (OTFs) were used for reconstruction. OTFs were computed using SoftWorx routines starting from the point spread function of 100 nm FluoSpheres (LifeTechnologies) matching the targeted wavelengths. Reconstruction filter settings used in all channels was set to 0.002. Absence of artifacts in 3D-SIM reconstructions was verified by employing different strategies. These include the recently developed SIM-Check software[11] which allowed us to verify or assure:

- negligible photobleaching during acquisition,
- negligible movement or drift of the sample,
- a strong modulation contrast,
- the absence of artifactual signatures in the fourier transform of the reconstructed data
- the presence of the first and second orders of the excitation modulation in the Fourier transform of the raw data.

Our approach to label and image the nucleoid by 3D-SIM was validated in our previous work [9].

For each channel a total of 255 images made of 17 different Z-planes separated by 125 nm each were acquired. Three different angles (-60°, 0° and +60°) as well as five phase steps were used to reconstruct 3D-SIM images using softWoRx v5.0 (Applied Precision Inc.). Final voxel size was 39.5 nm in xy and 125 nm in z for a final 3D stack volume of 40 x 40 x 2 μm^3. Multicolor TetraSpeck beads (100 nm in diameter, Invitrogen) were used to measure x, y and z offsets, rotation about the z-axis and magnification differences between fluorescence channels. These corrections were applied to the reconstructed images. The same beads were used to validate the reconstruction process ensuring a final resolution of ~120 nm in xy and ~350 nm in z [9]. Acquisition parameters were used as follows: DAPI, 5-10 ms exposure with 10% transmission of the 405 nm excitation line; mVenus, 10 ms exposure with 31.3% transmission of the 488 nm excitation line and HU-mCherry 10ms exposure with 31.3% transmission of the 568 nm excitation line.

For *widefield deconvolution microscopy* imaging, 25 Z-slices were acquired for each fluorescent channel with integration times of 10ms on the same optical setup. Experimental point spread functions (PSF) for each color was measured by imaging multicolor fluorescent beads (Tetraspeck, 100 nm diameter, Invitrogen) and used for aligning fluorescent channels. 3D raw data was deconvolved by 3D iterative deconvolution, employing the Quick Maximum Likelihood Estimator algorithm of Huygens software (Scientific Volume Imaging). Volumetric acquisitions using Multi Focus Microscopy were conducted on an Zeiss Axiovert 200 with custom optical elements inserted into the emission pathway as follows: a diffraction grating was conjugated through the microscope tube lens with the back focal plane of the objective (100×, 1.4 N.A.; Zeiss). The grating acts to direct the incoming fluorescent photons from distinct Z planes from the sample into nine central orders with ~65% efficiency. Then, a combination of a blazed grating and prism is used to correct for residual chromatic dispersion. Finally, an imaging lens forms the image of the nine imaging planes on a single EM–CCD detector (DU-897; Andor). Fluorescence excitation is provided with a 488 and 561 nm laser lines controlled by an acousto-optic tunable filter (AA Opto-Electronic) and directed to the objective of the microscope by a multiband dichroic mirror (LF405/488/561/635–4 × 4M-A-000; Semrock). MFM integration time is 25 ms. The nine imaging planes are separated by 209 and 225 nm in the mVenus and mCherry fluorescence channels, respectively. Calibration of the distance between planes, channels registration and alignment of different panels was made by scanning a sample of fluorescent beads (Invitrogen, 40 nm diameter) along the optical axis.

Single-particle tracking PALM imaging of ParA-mEos2 was performed on a home-built experimental setup based on a Zeiss Axiovert 200 by continuously acquiring between 14000 and 28000 images at ~100Hz (8ms acquisition time) under stroboscopic illumination with a 561 nm readout laser (4ms exposure per frame, Sapphire 561LP, 100mW, Coherent) and continuous illumination with a 405 nm laser for photo-activation (OBIS 405-50, Coherent). The readout laser intensity used for spt-PALM experiments was 1.2 kW/cm² at the sample plane. For the activation laser (405nm), the maximum power used for photo-activating mEos2 was 0.1W/cm². The intensity of the 405nm laser was modified during the course of the experiment to maintain the density of activated fluorophores constant while ensuring that only one protein was activated at a time in a single diffraction-limited spot. Acquisition was carried out until all mEos2 proteins were photo-activated and bleached. Fluorescent beads (TetraSpeck TM, Life Technologies) were used as fiducial marks to correct for sample drift during post-processing analysis.

Data analysis

3D-SIM data were analyzed using homemade routines written in Matlab (Mathworks, Inc, US). Each individual bacteria and nucleoid were automatically segmented using a 3D maximum entropy thresholding algorithm for binarization and minimum and maximum volume values for bacteria and nucleoids detection. The center of mass, long and short axis were computed on the binarized nucleoids and used to rotate the original nucleoid so that the major nucleoid axis was aligned with the x-axis. HDR, ParB foci and ParA patches were identified as 3D local maxima in each corresponding fluorescent channel and detected using a multi-directional derivation of the intensity scalar vector smoothed to avoid detection of noise. Nucleoids lengths and widths were measured by determining the full width at half maximum (FWHM) in the long and short axes of the nucleoid, and used to normalize the positions of 3D local maxima. To discard outliers from the distribution, histograms of width, height, and nucleoid mean intensities were fitted with a Gaussian function and only nucleoids included within twice the FWHM were selected for further analysis. 2D histograms of the positions of HDR, ParB foci or ParA patches in the nucleoids were constructed as follows: nucleoids lengths were segmented into windows (typically of 250 nm) for which the mean and the standard deviation (STD) of the number of local maxima are computed, another selection was then performed to keep only nucleoids that showed a number of local maxima falling within one standard deviation of the mean. For each window, the selected nucleoids were resampled 100 times using a bootstrapping method and local maxima positions orientation were randomized along the long, short and vertical axis of the nucleoids before computing 2D histograms. 2D histograms were generated by computing the 2D spatial distribution of number of local maxima weighted by their corresponding local voxel intensity in the 3D-SIM stack. The mean and STD of the histograms were calculated for each window from the 100 smoothed histograms built from the 100 bootstrapped samples. Note that for YZ histograms construction, solely ParB foci whose longitudinal coordinates lie within 60% of the nucleoid length were considered in order to avoid the contribution from ParB foci at the nucleoid poles.

Kymographs were computed following the same procedure but using a 100 nm running window with 20 nm steps. Kymographs were computed by integrating all maxima localizations within -0.5 to 0.5 over the short axis.

Single-particle tracking PALM analysis was performed using the Multiple-Target Tracing (MTT) software [12]. Single ParA-mEos2 localizations were linked to single tracks if they appeared in consecutive frames within a window of 478 nm. To account for fluorescent protein blinking or missed events, labeled ParA-mEos2 molecules were allowed to disappear for a maximum of 3 frames. The apparent diffusion coefficient was calculated from the mean-squared displacement (MSD) for each track with a minimum of nine frames using custom built routines written in Matlab.

Supplementary References

1. Sanchez, A. *et al.* Stochastic Self-Assembly of ParB Proteins Builds the Bacterial DNA Segregation Apparatus. *Cell Systems* **1,** 163–173 (2015).
2. Woldringh, C. L., Hansen, F. G., Vischer, N. O. E. & Atlung, T. Segregation of chromosome arms in growing and non-growing Escherichia coli cells. *Front. Microbiol.* **6,** (2015).
3. Cabrera, J. E., Cagliero, C., Quan, S., Squires, C. L. & Ding, J. J. Active transcription of rRNA operons condenses the nucleoid in Escherichia coli: Examining the effect of transcription on nucleoid structure in the absence of transertion. *J. Bacteriol.* **191,** 4180–4185 (2009).
4. Youngman, P. J., Perkins, J. B. & Losick, R. Genetic transposition and insertional mutagenesis in Bacillus subtilis with Streptococcus faecalis transposon Tn917. *Proc. Natl. Acad. Sci. U. S. A.* **80,** 2305–2309 (1983).
5. Bouet, J. Y., Bouvier, M. & Lane, D. Concerted action of plasmid maintenance functions: Partition complexes create a requirement for dimer resolution. *Mol. Microbiol.* **62,** 1447–1459 (2006).
6. Marceau, A. H. *et al.* Structure of the SSB–DNA polymerase III interface and its role in DNA replication. *EMBO J.* **30,** 4236–4247 (2011).
7. Diaz, R., Rech, J. & Bouet, J.-Y. Imaging centromere-based incompatibilities: Insights into the mechanism of incompatibility mediated by low-copy number plasmids. *Plasmid* (2015). doi:10.1016/j.plasmid.2015.03.007
8. Lemonnier, M., Bouet, J. Y., Libante, V. & Lane, D. Disruption of the F plasmid partition complex in vivo by partition protein SopA. *Mol. Microbiol.* **38,** 493–505

(2000).

9. Marbouty, M. *et al.* Condensin- and Replication-Mediated Bacterial Chromosome Folding and Origin Condensation Revealed by Hi-C and Super-resolution Imaging. *Mol. Cell* **59,** 588–602 (2015).

10. Fiche, J.-B. *et al.* Recruitment, Assembly, and Molecular Architecture of the SpoIIIE DNA Pump Revealed by Superresolution Microscopy. *PLoS Biol.* **11,** e1001557 (2013).

11. Ball, G. *et al.* SIMcheck: a Toolbox for Successful Super-resolution Structured Illumination Microscopy. *Sci. Rep.* **5,** 15915 (2015).

12. Serge, A., Bertaux, N., Rigneault, H. & Marguet, D. Dynamic multiple-target tracing to probe spatiotemporal cartography of cell membranes. *Nat. Methods* **5,** 687–694 (2008).
