# Supplementary figures and images for: Bacterial partition complexes segregate within the volume of the nucleoid
Source: Nat Commun. 2016 Jul 5;7:12107. doi: 10.1038/ncomms12107 (PMC4935973; doi:10.1038/ncomms12107)

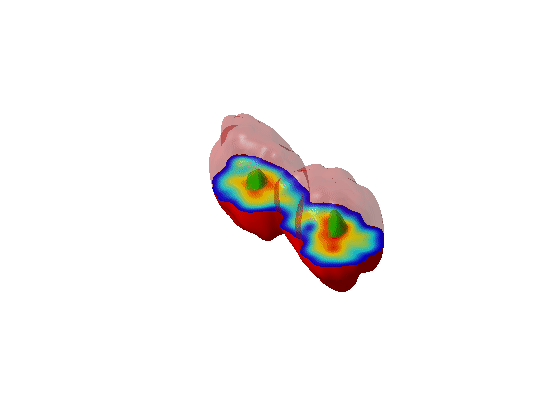

Supplement: Supplementary Movie 1 — 3D-SIM of an E. coli nucleoid (solid red) carrying a wild type mini-F plasmid with parBF tagged mVenus (green). Strain DLT3053/pJYB234. [file ncomms12107-s2.gif]

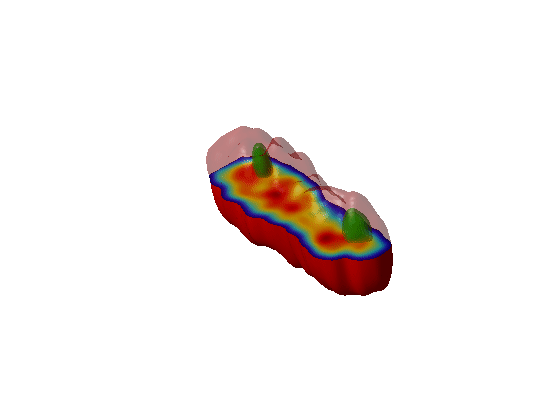

Supplement: Supplementary Movie 2 — 3D volume rotation of a B. subtilis nucleoid (solid red) with parBBsu tagged GFP (green) imaged in 3D-SIM. Strain HM671. [file ncomms12107-s3.gif]

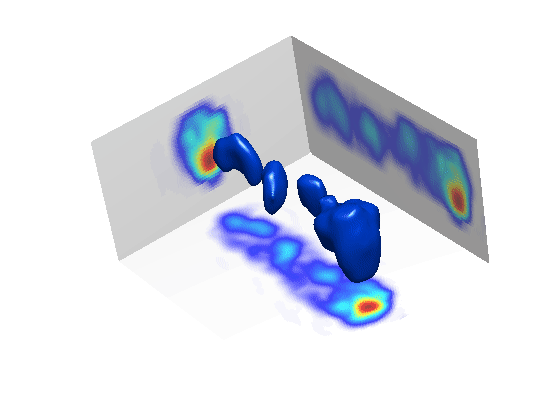

Supplement: Supplementary Movie 3 — 3D volume of ParAF (blue) with side projections of ParAF densities, imaged in 3D-SIM. Strain DLT3053/pJYB243. [file ncomms12107-s4.gif]

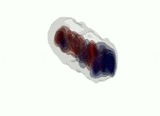

Supplement: Supplementary Movie 4 — 3D volume of an E. coli nucleoid (solid red) ParAF (blue) imaged in 3D-SIM. Strain DLT3053/pJYB243. [file ncomms12107-s5.gif]

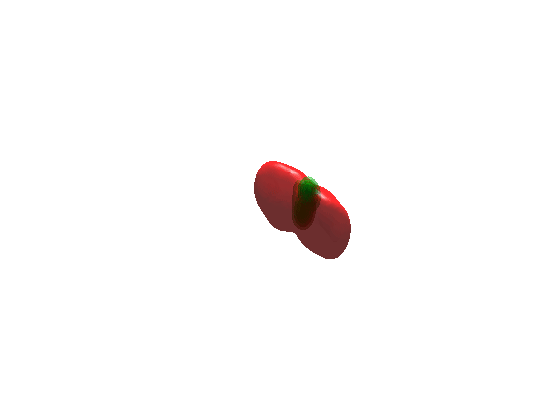

Supplement: Supplementary Movie 5 — 3D epifluorescence imaging of E. coli cells harboring F-plasmids lacking ParAF and with ParB tagged with mVenus. Strain DLT3053/pJYB263. [file ncomms12107-s6.gif]

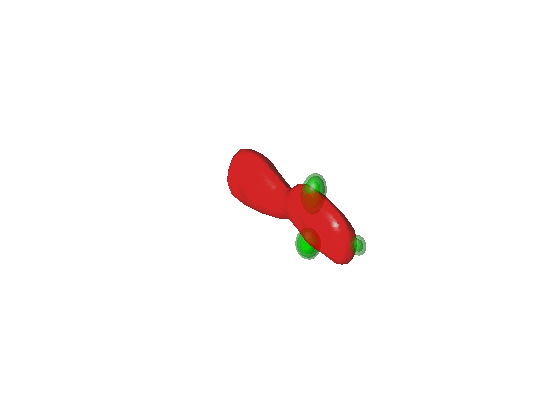

Supplement: Supplementary Movie 6 — 3D epifluorescence imaging of E. coli cells harboring F-plasmids lacking ParAF and with ParB tagged with mVenus. Strain DLT3053/pJYB263. [file ncomms12107-s7.gif]

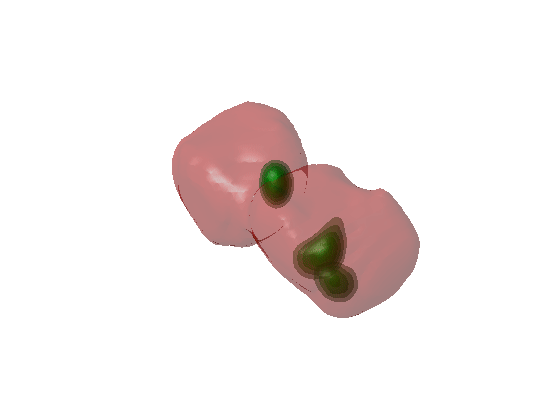

Supplement: Supplementary Movie 7 — 3D epifluorescence imaging of E. coli cells harboring F-plasmids with ParAF-K120Q, an allele in which the ParBF stimulation of ParAF's ATP hydrolysis is abolished, and with ParB tagged with mVenus. Strain DLT3053/pJYB277. [file ncomms12107-s8.gif]

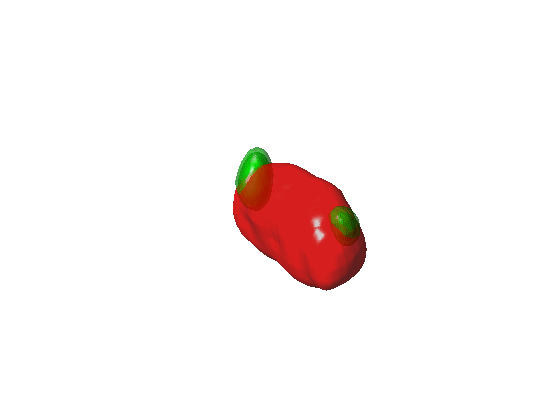

Supplement: Supplementary Movie 8 — 3D epifluorescence imaging of E. coli cells harboring F-plasmids with ParAF-K340A, an allele deficient in ns-DNA binding, and with ParB tagged with mVenus. Strain DLT3053/pJYB279. [file ncomms12107-s9.gif]

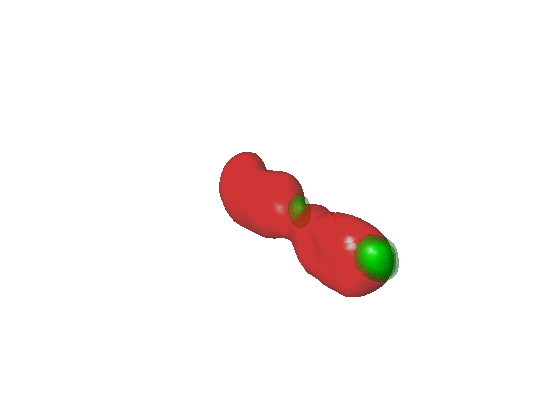

Supplement: Supplementary Movie 9 — 3D epifluorescence imaging of E. coli cells harboring F-plasmids with ParAF-K340A, an allele deficient in ns-DNA binding, and with ParB tagged with mVenus. Strain DLT3053/pJYB279. [file ncomms12107-s10.gif]

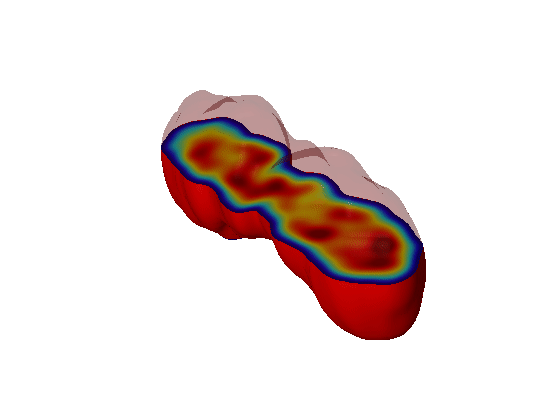

Supplement: Supplementary Movie 10 — 3D-SIM of an E. coli nucleoid (solid red). Strain DLT3053/pJYB234. [file ncomms12107-s11.gif]
